# Sensorimotor, language, and working memory representation within the human cerebellum

**DOI:** 10.1002/hbm.24733

**Published:** 2019-07-30

**Authors:** Reiko Ashida, Nadia L. Cerminara, Richard J. Edwards, Richard Apps, Jonathan C. W. Brooks

**Affiliations:** ^1^ School of Physiology, Pharmacology and Neuroscience University of Bristol Bristol UK; ^2^ Neurosurgery Department, Southmead Hospital North Bristol Trust Bristol UK; ^3^ Neurosurgery Department, Bristol Royal Hospital for Children University Hospitals Bristol NHS Foundation Trust Bristol UK; ^4^ Bristol Medical School University of Bristol Bristol UK; ^5^ School of Psychological Science University of Bristol Bristol UK

**Keywords:** cerebellum, fMRI, language, sensorimotor, working memory

## Abstract

The cerebellum is involved in a wide range of behaviours. A key organisational principle from animal studies is that somatotopically corresponding sensory input and motor output reside in the same cerebellar cortical areas. However, compelling evidence for a similar arrangement in humans and whether it extends to cognitive functions is lacking. To address this, we applied cerebellar optimised whole‐brain functional MRI in 20 healthy subjects. To assess spatial overlap within the sensorimotor and cognitive domains, we recorded activity to a sensory stimulus (vibrotactile) and a motor task; the Sternberg verbal working memory (VWM) task; and a verb generation paradigm. Consistent with animal data, sensory and motor activity overlapped with a somatotopic arrangement in ipsilateral areas of the anterior and posterior cerebellum. During the maintenance phase of the Sternberg task, a positive linear relationship between VWM load and activity was observed in right Lobule VI, extending into Crus I bilaterally. Articulatory movement gave rise to bilateral activity in medial Lobule VI. A conjunction of two independent language tasks localised activity during verb generation in right Lobule VI‐Crus I, which overlapped with activity during VWM. These results demonstrate spatial compartmentalisation of sensorimotor and cognitive function in the human cerebellum, with each area involved in more than one aspect of a given behaviour, consistent with an integrative function. Sensorimotor localisation was uniform across individuals, but the representation of cognitive tasks was more variable, highlighting the importance of individual scans for mapping higher order functions within the cerebellum.

## 1 INTRODUCTION

The cerebellum is critically involved in the coordination of reflex and voluntary movements, the postural base required for such movements and the learning of new motor skills, for review see (Ito, 1984). An increasing body of evidence also indicates that its role extends to cognition and affect (Keren‐Happuch, Chen, Ho, & Desmond, 2014; Koziol et al., 2014; Leiner, Leiner, & Dow, 1986; Lesage, Hansen, & Miall, 2017; Schmahmann & Sherman, 1998; Stoodley, 2012; Stoodley, 2014; Strick, Dum, & Fiez, 2009). To gain a full understanding of the role the cerebellum plays in such a diverse range of behaviours it is important to first establish whether such functions are regionally compartmentalised, and whether different facets of a given behaviour (e.g., sensory and motor components of somatic behaviour) are represented in the same spatial area.

It has long been known from animal studies that somatotopically organised maps are present within the cerebellum. For instance, direct electrophysiological mapping has revealed in a range of species a dual representation of the upper and lower limbs in the anterior and posterior lobes of the cerebellar cortex (Atkins & Apps, 1997; Ekerot & Larson, 1979; Garwicz, 1997; Jorntell, Ekerot, Garwicz, & Luo, 2000; Pardoe & Apps, 2002; Pijpers, Apps, Pardoe, Voogd, & Ruigrok, 2006; Snider & Stowell, 1944). Generally speaking, noninvasive neuroimaging studies suggest a corresponding somatotopy in the human cerebellum (Grodd, Hulsmann, Lotze, Wildgruber, & Erb, 2001; Kuper et al., 2012; Rijntjes, Buechel, Kiebel, & Weiller, 1999). Distinct topographical regions have also been found for higher order (‘cognitive’) functions in the human cerebellum. For example, studies employing positron emission tomorgraphy and functional magnetic resonance imaging (fMRI) during language tasks have identified in right‐handed subjects the involvement of right Lobule VI and Crus I in such tasks (Frings et al., 2006; Jansen et al., 2005; Petersen, Fox, Posner, Mintun, & Raichle, 1989; Stoodley, Valera, & Schmahmann, 2012), whereas working memory paradigms have been shown to activate bilateral regions of Lobules VI/Crus I and VII and right VIIIA (Chen & Desmond, 2005a; Desmond, Gabrieli, Wagner, Ginier, & Glover, 1997; Guell, Gabrieli, & Schmahmann, 2018; Kirschen, Chen, & Desmond, 2010; Kirschen, Chen, Schraedley‐Desmond, & Desmond, 2005).

There is, however, disagreement in the neuroimaging literature regarding topographical organisation of somatic and cognitive functions in the human cerebellum. In relation to motor mapping, some studies report bilateral activation in the anterior and posterior cerebellum with enhanced activation on the side ipsilateral to the moving body part (Grodd et al., 2001; Kapreli et al., 2007; Kuper et al., 2012; Nitschke, Kleinschmidt, Wessel, & Frahm, 1996; Rijntjes et al., 1999), while others have reported only ipsilateral activation (Schlerf, Verstynen, Ivry, & Spencer, 2010; Spencer, Verstynen, Brett, & Ivry, 2007; Stoodley et al., 2012). Inconsistencies in relation to sensory mapping have also been found, with some studies reporting ipsilateral cerebellar activation (Fox, Raichle, & Thach, 1985; Tempel & Perlmutter, 1992; Trulsson, Francis, Bowtell, & McGlone, 2010; Wiestler, McGonigle, & Diedrichsen, 2011) while others report bilateral cerebellar activation (Golaszewski et al., 2006). Discrepancies are also present in relation to mapping cognitive function: bilateral activation of Lobule VI (Keren‐Happuch et al., 2014; Stoodley & Schmahmann, 2009) and Lobules VIII (Hautzel, Mottaghy, Specht, Muller, & Krause, 2009; Kuper et al., 2016) has been reported for language and working memory tasks, respectively.

Most imaging studies have used single behavioural paradigms (e.g., sensorimotor or working memory or language) to map cerebellar topography (e.g., Frings et al., 2006; Grodd et al., 2001; Rijntjes et al., 1999) which limits the ability to map cerebellar functional topography particularly when examining behaviours which may activate common areas of the cerebellum. Nonhuman animal studies (e.g., Bauswein, Kolb, Leimbeck, & Rubia, 1983; Proville et al., 2014; Snider & Stowell, 1944) and imaging studies have provided some evidence that sensorimotor tasks map to overlapping regions within the cerebellum (Fox et al., 1985; Stoodley et al., 2012). Meta‐analysis of imaging data within the cognitive domain has also been shown to overlap (Keren‐Happuch et al., 2014; Stoodley & Schmahmann, 2009). However, the pooling of studies by meta‐analyses has a number of limitations: results are influenced by the combination of different sample sizes, the effects of scanning at different magnetic field strengths, differences in analysis methodologies and anatomical heterogeneity. To overcome these issues, imaging studies are required that investigate a range of sensorimotor and cognitive tasks within the same subjects. To date, two previous studies have attempted to do this. Stoodley et al. (2012) found in a cohort of nine subjects that nonoverlapping areas of the cerebellum were activated during overt movement (‘sensorimotor’) and cognitive tasks with differing task demands (language, working memory, spatial, and affective tasks). An area of overlap was found between language and working memory tasks; however, this was not confirmed by a formal conjunction analysis. Most recently, Guell et al. (2018) reported on 787 subjects studied as part of the Human Connectome Project (HCP, Van Essen, et al., 2013) that a nonoverlapping representation for cognitive tasks involving working memory and language was present in the cerebellum. Therefore, while spatial overlap of cerebellar activity in response to somatosensory task might be expected based on previous studies, spatial overlap of cognitive tasks remains to be substantiated.

The present study therefore images the spatial patterns of cerebellar activation in relation to a range of different tasks within a cohort of individuals. This has the additional advantage that intersubject variability can be determined. There are a number of studies which have assessed this for cerebral cortical activity (Kherif, Josse, Seghier, & Price, 2009; Miller, Donovan, Bennett, Aminoff, & Mayer, 2012; Seghier & Price, 2016; Seghier & Price, 2018). For example, Miller et al. (2012) found that patterns of fMRI activation in the cerebral cortex during a memory retrieval task differed between individual subjects from the pattern of activity derived from group analysis—which was attributed to variation in cognitive processing and encoding style. However, to our knowledge, no previous cerebellar imaging study has examined this important issue. Therefore, the aims of our study, using high‐resolution cerebellar optimised whole‐brain fMRI in a cohort of 20 subjects, were to: (a) map within the same subjects somatic (limb and articulator) and cognitive [language and verbal working memory (VWM)] representation in the human cerebellum; (b) assess the degree of spatial overlap of different facets of somatic and cognitive function using separate sensory and motor tasks; language and VWM tasks, respectively; and (c) determine the degree of variability between subjects in these spatial maps. To independently examine motor and sensory processes, we used an externally paced motor task and ‘passive’ vibrotactile stimulation targeting the upper and lower limbs; for the cognitive domain, we chose tasks that ostensibly test different domains—a verb generation paradigm (language) and the Sternberg working memory task, though both presumably rely on VWM.

## 2 METHODS

### 2.1 Participants

The study was approved by the University of Bristol, Faculty of Medicine and Veterinary Science Committee for Ethics (FMV‐462). Subjects were drawn from the University staff and student population. All participants provided written informed consent to take part in the study, which followed the ethical principles of the Declaration of Helsinki (World Medical Association, 2013). Twenty (14 females) right‐handed healthy adult participants were recruited. One participant was excluded from the Sternberg VWM task and language tasks as they were later found to be dyslexic. Thus, 20 subjects completed the motor and vibrotactile paradigms, and 19 subjects performed the language and Sternberg paradigms. Their median age was 27.5 and the age range was 23–44 years. All participants spoke English as their first language. None of the participants had any history of neurological conditions or contraindications to MRI. To better characterise the study sample, and confirm that all subjects lay within the normal range, all participants completed the comprehension, digit span, letter number sequencing, and arithmetic subtests of the Wechsler Adult Intelligence Scale, version (WAIS‐IV; Wechsler, 2011), which broadly assessed VWM and language skills. Motor function was assessed with the grooved pegboard (Lafayette Instrument Company, Lafayette, IN) by recording the time required to complete the task. The participants' handedness was confirmed using the Edinburgh Handedness Inventory (Oldfield, 1971).

### 2.2 Tasks

Four tasks were performing during scanning. Visual/auditory presentation and stimulus timing were controlled by a computer running Presentation software (Neurobehavioral Systems, Albany, CA).

#### 2.2.1 Motor

Subjects were instructed to move their right fingers or right toes in time with a visual cue. Following a 1‐s cue ‘*’ either ‘finger’ or ‘toe’ were presented on screen at an irregular rate. There were 20 blocks in total, consisting of 10 blocks with finger movement (repeated flexion extension of metacarpophalangeal and interphalangeal joints) and 10 blocks with toe movement (repeated flexion extension of metatarsophalangeal and interphalangeal joints). Each block had a duration of 9 s. An ABC design was used with finger movement followed by, or preceded by, toe movement followed by 12 s of rest. The order of blocks was randomised.

#### 2.2.2 Sensory

Vibrotactile stimuli were delivered to the right index finger and the right first toe or to both simultaneously, using an MR‐compatible piezoelectric stimulators (Piezo Tactile Stimulator PTS‐C2; Dancer Design, St. Helens, UK) driven at 150 Hz, to preferentially stimulate Pacinian corpuscles (Morioka & Griffin, 2005). The stimulus amplitude was adjusted to ensure it was perceived as isointense at the two sites, and clearly noticeable. In total there were 10 ‘finger’, 10 ‘toe’, and 5 ‘both’ stimulation blocks. To maintain attention and minimise adaptation during stimulation, stimuli were ‘chirped’ with variable duration and gaps (Ai, Oya, Howard, & Xiong, 2013; Nelson, Staines, Graham, & McIlroy, 2004). Following each block, subjects were prompted via a visual instruction to press a button (MRI‐compatible button box, Lumina LP‐400; Cedrus Corporation, San Pedro, CA) if they had detected a brief (single chirp) switch between, for example, stimulating the finger to the toe (or vice versa) during stimulation. All blocks were presented in random order, with each lasting for 9 s, preceded by a 1 s cue, followed 1 s later by the switch detection question, followed by 11 s of rest.

#### 2.2.3 Language/speech motor

During scanning, nouns or non‐words (non‐words list generated from the ARC Non Word Database; Rastle, Harrington, & Coltheart, 2002) were delivered to subjects, via MRI compatible headphones (Model S14 Sensimetrics Corporation, Malden, MA), who performed one of five different conditions (see below). To minimise background scanner, noise subjects had memory foam padding placed over the ears. Prior to commencing the language task, we assessed whether subjects could hear stimuli over the echo planar imaging (EPI) sequence, adjusting the volume as necessary. Each block lasted 24 s, and commenced with a 3 s visual instruction:

1. ‘Listen to the noun, generate verb and say aloud’, for example, *ball* is heard, say verb ‘*kick*’.
2. ‘Listen to the noun, generate verb in your head’, for example, *ball* is heard, think verb ‘kick’.
3. ‘Just listen to the noun’, for example, *ball* is heard.
4. ‘Listen to non‐word and repeat aloud’, for example, *dulf* is heard, say ‘dulf’.
5. ‘Just listen to non‐word’, for example, *dulf* is heard.

There were 20 blocks in total, four repetitions of each type. Seven audio stimuli of the same type were played per block, with each noun or non‐word played lasting less than 1 s. Audio stimuli were presented every 3 s, leaving approximately 2 s to perform the instructed task. Eighty‐four commonly occurring nouns and 56 non‐words were shuffled and used for presentation, without repetition. An ABCD design was used including rest blocks with duration of 24 s. Each loop consisted of at least one rest block and either three trial blocks or two trial blocks and an additional rest block.

#### 2.2.4 VWM (Sternberg task)

This task used an event‐related design, with timings chosen to minimise collinearity of regressors (Cairo, Liddle, Woodward, & Ngan, 2004). Consonants were displayed on a screen during the encoding phase, in keeping with other fMRI studies using the Sternberg task (Chen & Desmond, 2005a; Kirschen et al., 2010). A variable working memory load was used with either two, four, six, or eight letters displayed during each trial. Subjects were instructed that they should memorise the letter sequence displayed on screen for 4 s (encoding phase) and keep the letters in mind during the maintenance phase lasting 3, 4, or 5 s by rehearsing the letters subvocally. Subsequently, during the recall phase a single letter probe was displayed for 2 s, which was either present or absent in the letter sequence, and subjects' responses (match or mismatch) recorded using a button box. There were 28 trials in total, during which each of the four working memory loads were presented seven times. The response accuracy and reaction time for the recall phase was recorded. Stimulation was jittered to allow more efficient sampling of the haemodynamic response function (Ollinger, Corbetta, & Shulman, 2001).

### 2.3 MRI acquisition

Scanning was performed on a Siemens 3T Skyra system (Erlangen, Germany) using a receive‐only 32‐channel head coil. Subjects' heads were secured with memory foam pads to minimise movement artefacts. To record cardiac and respiratory waveforms during fMRI scans, a pulse oximeter and respiratory bellows were attached to the subject and data recorded using an MP150 system (BIOPAC Systems Inc., Goleta, CA). Following acquisition of localiser images, a sagittal T1‐weighted structural scan (magnetization prepared rapid gradient echo) with AP phase encoding direction, covering the cerebrum, cerebellum, and brainstem was acquired. The parameters used were 1.0 × 1.0 × 1.0 mm^3^ voxel size, echo time (TE)/repetition time (TR) 2.99/2,300 ms, flip angle 9°, field of view (FOV) 224 × 218 mm^2^, bandwidth 240 Hz/Px and generalised auto‐calibrating partially parallel acquisitions (GRAPPA, Griswold et al., 2002) acceleration factor 2. Subsequently, functional imaging data were acquired with an EPI sequence, aligned such that the axial slices were perpendicular to the base of the fourth ventricle (thus, roughly parallel to the horizontal fissure of the cerebellum). Scans were acquired during the four tasks (see above) with the following parameters: in‐plane voxel size 1.8 × 1.8 mm^2^, slice thickness 3.5 mm, TE/TR 30/3,000 ms, flip angle 80°, FOV 170 × 170 mm^2^, bandwidth 1,646 Hz/Px, phase encoding anterior to posterior and GRAPPA acceleration Factor 2. We chose to use anisotropic voxels as a trade‐off between retaining high in‐plane resolution (1.8 × 1.8 mm^2^) across the approximately dorso‐ventral arrangement of the cerebellar lobules, while retaining whole brain coverage within a reasonable TR. Following the fMRI task scans and for the purpose of EPI distortion correction, a dual echo gradient‐echo field map was acquired with 3 × 3 × 3 mm^3^ resolution, FOV 192 × 192 mm^2^ and TE1/TE2/TR = 4.92/7.38/520 ms.

### 2.4 Analysis

Functional imaging data were analysed using the FSL software package (FMRIB's Software Library, http://fsl.fmrib.ox.ac.uk, v5.0.9.1). Each subject's structural scan was brain extracted using a custom routine utilising the VBM8 package in SPM software to segment the brain into grey matter, white matter, and cerebrospinal fluid (CSF), and the sum of the three components used to define the brain's boundary. Subsequently, fMRI data were adjusted for EPI distortions using field‐maps derived using (FMRIB's utility for geometrically unwarping EPIs, Jenkinson, 2003), motion corrected using (motion correction using FMRIB's linear image registration tool, Jenkinson, Bannister, Brady, & Smith, 2002), high‐pass temporally filtered (cut‐off 90 s) and spatially smoothed with a kernel of 3 mm full‐width half maximum. The applied smoothing kernel was isotropic and was chosen as a compromise between the need to have data that are spatially smooth (required by random field theory, used for statistical inference, Worsley, Evans, Marrett, & Neelin, 1992) while retaining the ability the observe small regions of activity within the cerebellum. Spatial normalisation/coregistration for later group analysis was performed at this stage, with boundary‐based registration (Greve & Fischl, 2009) used to map each subject's functional data to their structural scan, and nonlinear registration using (FMRIB's non‐linear image registration tool, part of FSL) to register structural scans to the 2 mm resolution sixth generation nonlinear Montreal Neurological Institute (MNI) brain template (Grabner et al., 2006) with 5 mm warp field control point spacing.

Model estimation was performed using FEAT (FMRIB's Expert Analysis Tool, the general linear model in FSL software), which used information about the timing of stimuli (onset, duration, weight) and a canonical hemodynamic response function to predict brain responses. Effects due to temporal autocorrelation of the acquired time series data, which can invalidate assumptions of normality, were minimised by prewhitening (Woolrich, Ripley, Brady, & Smith, 2001). It has been demonstrated that the cerebellum, like the brainstem, suffers from issues relating to increased physiological noise, for example, due to pulsatile flow of CSF through the fourth ventricle (Brooks et al., 2008; Brooks, Faull, Pattinson, & Jenkinson, 2013; van der Zwaag, Jorge, Butticaz, & Gruetter, 2015). Hence, a physiological noise model, which attempts to model signal fluctuation in the fMRI time series produced by cardiac and respiratory processes (Brooks et al., 2008) was incorporated into the GLM. While task‐related activity was determined at the first (subject) level for simple contrasts versus rest, contrasts between conditions (e.g., main effects, parametric modulation) were estimated at the second (group) level on which results are based.

Group‐level inference was performed using univariate statistics and a mixed effects model within FEAT. Given recent concerns around the use of cluster‐based statistics (Eklund, Nichols, & Knutsson, 2016), we used a stringent approach to control for false positives. Statistical inference was performed using an initial cluster forming threshold of *Z* > 3.09, and a cluster‐based corrected significance level of *p* < .05 used for reporting (unless stated otherwise). This has the effect of increasing the specificity of our findings by (a) only considering voxels that meet a more stringent level of significance (at the voxel level), while (b) still correcting for family wise error using cluster‐based thresholding in the context of a mixed effects model. Following initial whole‐brain analyses, given our a priori hypotheses around the cerebellum and its involvement in the chosen tasks, cerebellar activity was assessed within an anatomical mask. The mask was based on a probabilistic cerebellar atlas (Diedrichsen, Balsters, Flavell, Cussans, & Ramnani, 2009), which was thresholded at 30%, and applied to data prior to statistical inference. Resultant statistical maps were interrogated using AUTOAQ (part of FSL) which uses probabilistic atlases of the cortical, subcortical, and cerebellar structures to determine the location of activated clusters, and the reported spatial locations confirmed by manual comparison to brain atlases.

At the second level, the following contrasts were generated to assess group activity:

1. *Motor*—(a) fingers > toes and (b) toes > fingers.
2. *Sensory* —(a) finger > toe and (b) toe > finger.
3. *Language*—The following contrasts versus rest were averaged: (a) verb generation aloud, (b) verb generation quietly (subvocal), (c) listen to nouns, (d) listen to and repeat non‐words, and (e) listen to non‐words. To isolate language processing and speech motor specific responses, the following contrasts between conditions were estimated: [speech motor 1 (SM1)] listen to nouns, generate verbs aloud minus generate verbs covertly (a *speech motor* contrast, due to vocalisation in the first condition, both contain a language component), [speech motor 2 (SM2)] listen to and repeat non‐words aloud minus listen to non‐words (a *speech motor* contrast, due to vocalisation in the first condition, neither contain a language component), (L1) listen to nouns, generate verbs covertly minus listen to nouns (a *language* contrast, silent language production in the first, but not the second condition) and (L2) listen to nouns, generate verbs aloud minus listen to and repeat non‐words (a *language* contrast, overt language production in the first condition, repetition of non‐words in the second condition). The similarity between conditions was determined using a conjunction analysis (Nichols, Brett, Andersson, Wager, & Poline, 2005).
4. *VWM*: To demonstrate activity associated with each working memory load simple averages versus rest were created for each of the four conditions (two, four, six, and eight letters) at encoding and maintenance. To test for brain regions whose activity increased linearly with increasing working memory load during encoding and maintenance, two further contrasts modelled a linear increase in activity using the following contrast vector [−3, −1, 1, 3] for memory loads two, four, six, and eight letters, respectively. As the recall condition necessarily contained a motor response, this period was modelled separately with a nuisance regressor and not considered further. For the Sternberg working memory task, results and discussion are based on the linear parametric contrasts.

To examine overlapping activity between different contrasts within or between paradigms, we used a conjunction analysis which utilised a cluster forming threshold of *Z* > 3.09 and corrected significance level of *p* < .05 (Eklund et al., 2016). To determine the degree of agreement between subjects within each paradigm, we created frequency maps (Brooks, Zambreanu, Godinez, Craig, & Tracey, 2005). Briefly, we took activation maps determined at the individual level with a cluster forming threshold of *Z* > 2.3 and corrected cluster significance of *p* < .05 and transformed them into space of the MNI standard brain. The spatially transformed statistical maps were then binarised and added together, such that the maximum intensity for any given voxel would be 20—indicating that all 20 subjects activated this particular region. We chose a pragmatic approach to visualising these data, setting a minimum threshold of five, that is, at least five subjects activated the voxels shown. Other approaches to determining consistency of activity have been proposed (Seghier & Price, 2016), but we believe our approach is likely to be conservative as anatomical differences will minimise overlap.

## 3 RESULTS

### 3.1 Behavioural task performance

All 20 participants underwent a set of neuropsychological tests as shown in Table S1, Supporting Information. Performance in tests of arithmetic (mean: 12.6, *SD*: 2.52), comprehension (mean: 11.8, *SD*: 2.02) was higher than normative data provided by WAIS‐IV, whereas the performance for letter number sequencing (mean: 6.3, *SD*: 0.86) was lower compared to the normative data. There were no differences in performance at digit span (assessment of working memory, mean: 10.3, *SD*: 2.39) and pegboard completion time (assessment of motor function) compared to normative data.

Performance (percentage correct) and reaction time (milliseconds) were assessed for the Sternberg test of VWM completed in the scanner (*N* = 19). A repeated measures analysis of variance (ANOVA) with Greenhouse–Geisser correction demonstrated a main effect of task difficulty on performance (*F*(1.816,32.693) = 16.316, *p* < .001). Post hoc tests with Bonferroni correction found that this was driven solely by reduced performance at highest load (8), *p* < .05. Reaction time data met assumptions of sphericity (Mauchly's test *p* = .695): a repeated measures ANOVA demonstrated a main effect of task difficulty on reaction time (*F*(3,54) = 14.39, *p* < .001). Linear regression modelling of the effect of increasing load on measured reaction time revealed a significant linear trend with slope 20.76 ms/item, *R* ^2^ = .2280 (*p* < .0001). The results demonstrate the expected deterioration in performance with increasing working memory load, as shown in the original report (Sternberg, 1966).

### 3.2 BOLD activation during somatic tasks

**Figure 1:**
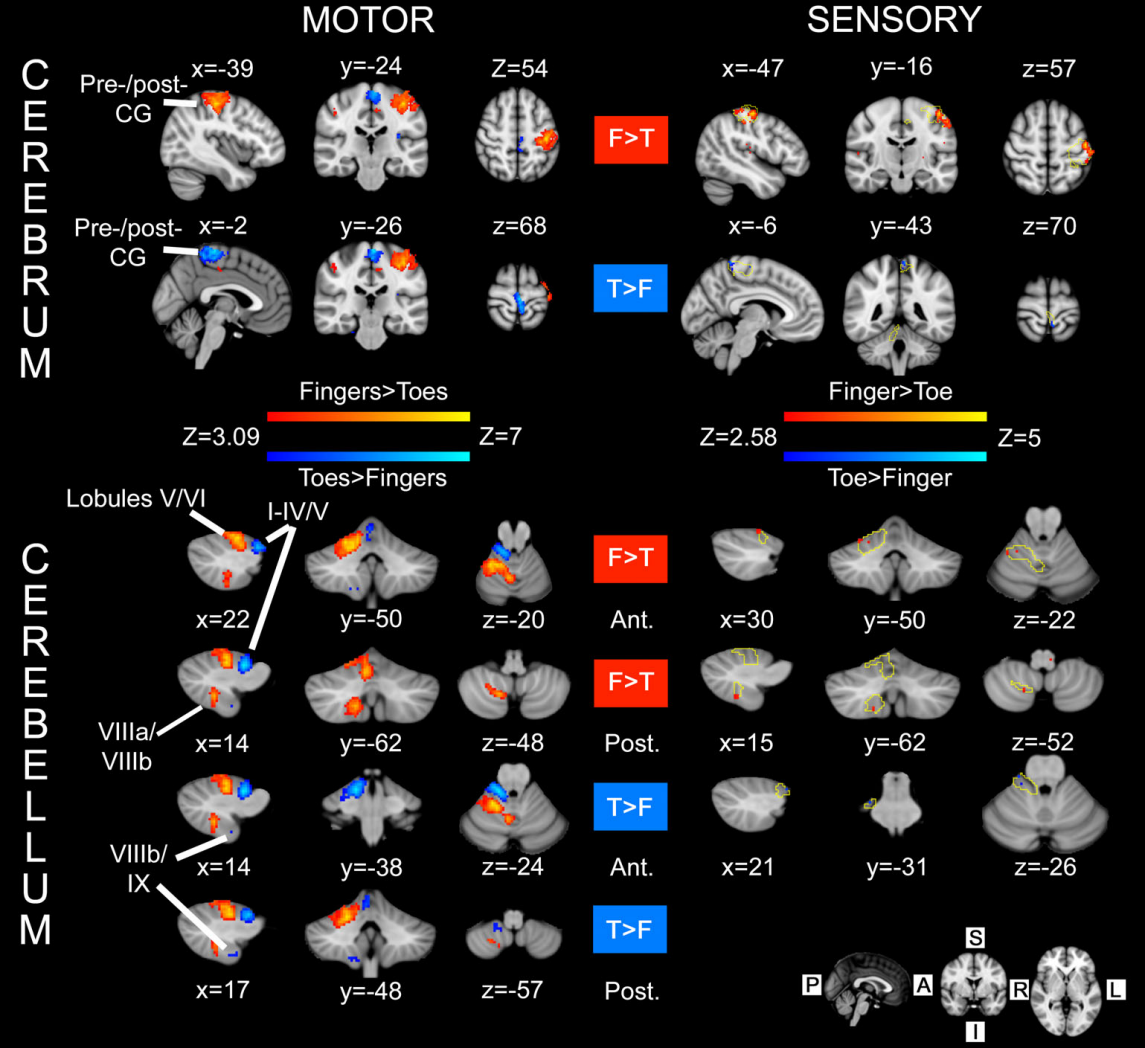
Sensorimotor integration in the cerebrum and cerebellum (*N* = 20). For both tasks (motor and sensory), the contrast between hand/finger > foot/toe is shown in red‐yellow colours, and foot/toe > hand/finger is shown in blue‐light blue colours. ‘Motor’: activity within the cerebrum and cerebellum in response to an externally paced movement task. Statistical maps reflect differences in activity in response to movement of the right hand and right foot (performed separately). ‘Sensory’: results of vibrotactile stimulation at 150 Hz of the right index finger and large toe on the right foot with MRI‐compatible piezoelectric tactile stimulators. Activity is shown overlaid on top of the corresponding motor maps (yellow outline). Convergence of sensorimotor input/output is found at the levels of the cerebrum (postcentral gyrus) and cerebellum (toes = Lobules I–IV and fingers = Lobule V). The anatomical level of each section is shown in Montreal Neurological Institute (MNI) coordinates (in mm) beside each image, corresponding to the location of the voxel with highest *Z*‐value for that contrast [see contrast labels: RED for finger(s) > toe(s), = ‘F > T’; BLUE for toe(s) > finger(s) = ‘T > F’]. Labels anterior/posterior: the listed coordinates refer to activity within anterior/posterior cerebellar lobes, respectively. Motor activity was assessed with cluster forming threshold *Z* > 3.09 and cluster corrected significance *p* < .05. Activity in response to vibrotactile stimulation for the cerebellum was obtained with an uncorrected significance threshold of *p* < .005 [Color figure can be viewed at http://wileyonlinelibrary.com]

Although our focus was the cerebellum, we examined activity in other brain regions associated with sensorimotor and cognitive tasks in order to evaluate the robustness of our paradigms for testing these functions. In response to visual instruction to move the right fingers or toes at an irregular rate, increased blood oxygenation level dependent (BOLD) activity occurred in the primary and supplementary motor cortices and occipital lobe (visual cortex). When directly contrasting conditions finger movement > toe movement, activity was observed in the hand area of the contralateral (to side of movement) sensorimotor cortex (Figure 1, Table S2, Supporting Information). The reverse contrast (toes > finger) gave rise to activity on the midline, corresponding to the foot area of the sensorimotor cortex (Figure 1, Table S3, Supporting Information). The same contrasts were run for cerebellar masked data (see Section 2 for details) and revealed a clear ipsilateral somatotopical arrangement, with the toes > fingers contrast associated with activity in Lobules I–IV of the anterior lobe and Lobules VIIIb and IX of the posterior lobe. By comparison, activation for the fingers > toes contrast was found in ipsilateral Lobules V and VI and Lobules VIIIa/b. Midline activation in Vermis VI and VIII was also found for the fingers > toes contrast.

Activity in response to sensory (vibrotactile) stimulation was statistically much weaker than for the motor paradigm and only the contrast finger > toe gave rise to activity with our adopted statistical threshold (*Z* > 3.09, cluster corrected *p* < .05). Consequently, we reduced the statistical significance threshold to *p* < .005 (uncorrected), to examine whether a pattern of activity consistent with the known ascending pathways to the sensory cortex could be observed. By using the contrast finger > toe, activity was observed in the contralateral (left) sensorimotor cortex (shown in red‐yellow on the right side of Figure 1, Table S4, Supporting Information), primarily in the postcentral gyrus, which lay within the activation area for the corresponding motor contrast (fingers > toes, yellow contour). For the toe > finger sensory contrast, activity (blue‐light blue in Figure 1, Table S5, Supporting Information) laid within the activation boundary for the toes > fingers motor contrast (yellow contour) near the midline postcentral gyrus.

As in the cerebrum, activity due to sensory stimulation and motor tasks overlapped in the cerebellum. Passive sensory stimulation of the index finger (ipsilateral Lobule V, contrast finger > toe) and big toe (ipsilateral Lobules I–IV, contrast toe > finger), produced activity which overlapped that from movement of the fingers and toes (see bottom half of Figure 1). Activity due to sensory stimulation and motor tasks also overlapped within the ipsilateral posterior cerebellar lobe, but this time only for the finger > toe contrast in right Lobule VIIIa/b.

### 3.3 Activation during language tasks

**Figure 2:**
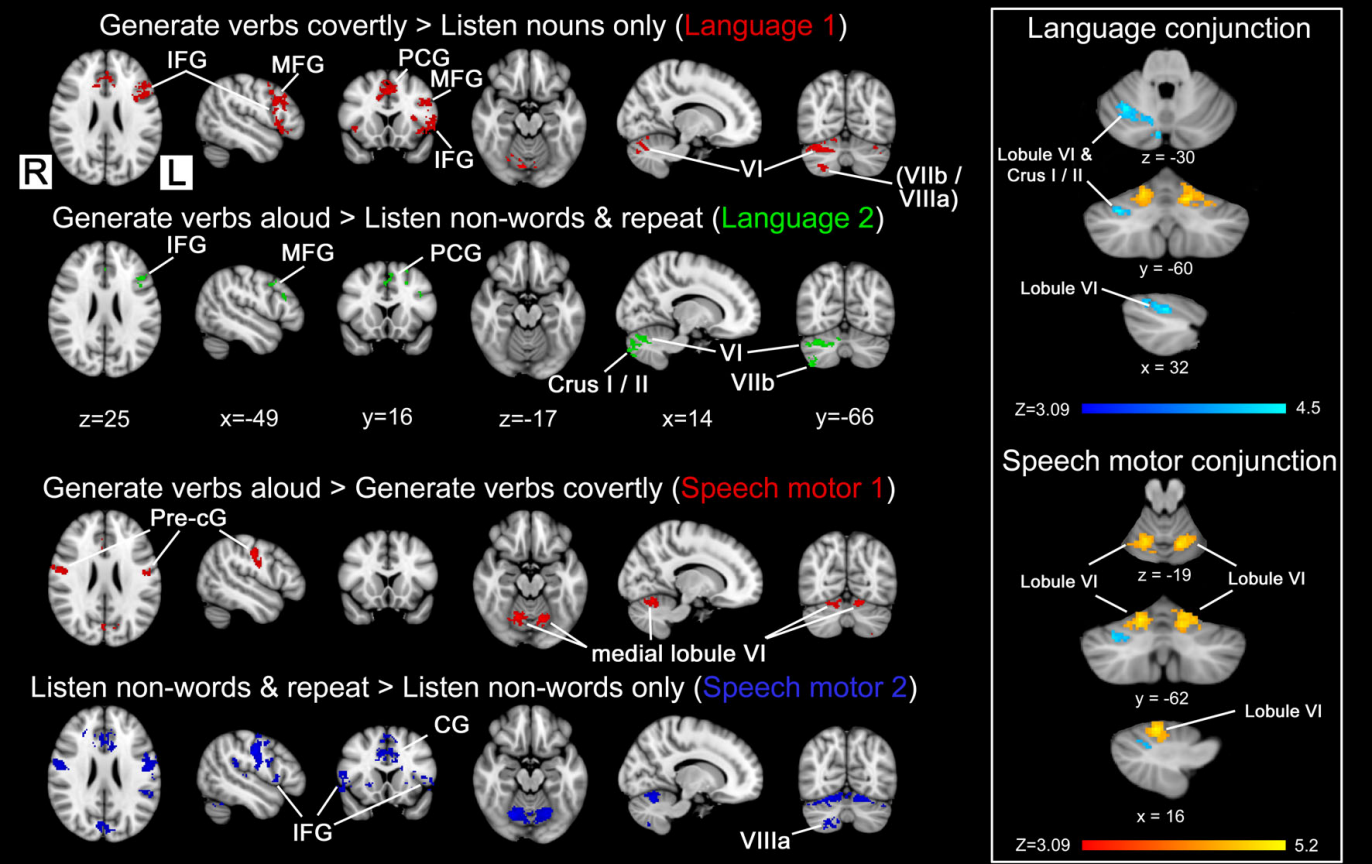
Language and speech motor contrasts and associated conjunctions for the language task (*N* = 19). Contrasts were used to isolate the different parts of the language task and are shown in red, green, and blue. The conjunction of language contrasts (L1 + L2) is blue‐light blue and speech motor contrasts (SM1 + SM2) is red‐yellow, shown for the cerebellum only. Broca's area and the anterior cingulate gyrus remained after subtracting conditions which were primarily associated with just auditory activity (i.e., Contrast L1) or auditory activity plus articulatory movement (i.e., Contrast L2). Activity for the different contrasts and conjunctions were determined using a cluster forming threshold of *Z* > 3.09 and cluster corrected *p* < .05. Montreal Neurological Institute (MNI) coordinates (in mm) are shown for the respective sections. CG, cingulate gyrus; IFG, inferior frontal gyrus; MFG, middle frontal gyrus; PCG, paracingulate gyrus; pre‐cG:, precentral gyrus [Color figure can be viewed at http://wileyonlinelibrary.com]

Contrasts between each of the five conditions and rest are presented in Figure S1, Supporting Information. All blocks involved auditory presentation of nouns or non‐words and produced activity in auditory cortex (superior temporal gyrus, cf. Rauschecker & Scott, 2009). More extensive involvement of the left inferior frontal gyrus (including pars opercularis and pars triangularis) was observed when the subject was asked to generate a verb associated with the heard noun or repeat a non‐word, compared to the listen only conditions. A more striking difference was observed in the cerebellum, with tasks involving vocal and silent speech producing activity within right and left Lobule VI extending into Crus I. To explore these differences, a series of contrasts were computed and are shown in Figure 2.

The contrasts (Language 1, L1) *generate verbs covertly* > *listen to nouns* and (Language 2, L2) *generate verbs aloud > listen to non‐words and repeat*, were designed to help isolate activity related to the verb generation component of the task while controlling for articulatory movement (present for both conditions in Contrast L2) and auditory presentation (present in all conditions)—see Figure 2. Contrast L1 produced activity which was primarily left lateralised in the cerebrum, with activity observed in the left inferior frontal gyrus, middle frontal gyrus, insula, posterior division of the superior temporal gyrus, and anterior cingulate cortex (see Table S6, Supporting Information). Contrast L2 generated less extensive activity that nonetheless still overlapped with Contrast L1 (see Figure 2, Table S7, Supporting Information). Focusing on the cerebellum, these language contrasts produced activity that was primarily right lateralised (see Table S7, Supporting Information). For Contrast L1, the largest cluster was found in right Lobule VI and Crus I, extending into Crus II and Vermis VI. The voxel with the highest *Z*‐score was found in Lobule VI. A smaller cluster was found in right Crus II, Lobules VIIb and VIIIa and in the left Lobule VI and Crus I. Contrast L2 was also localised to the right cerebellar hemisphere. The largest area of activation was found in the right Crus II extending into Crus I and Lobule VI. Similar to Contrast L1, right‐sided activity was found in Lobules VIIb and VIII. In the left cerebellar hemisphere, the L2 contrast produced activity within Lobules VI, VIIb, and VIII and Crus I. Conjunction analysis revealed a significant overlap between Conditions L1 and L2 within right cerebellar Lobule VI and Crus I (see right‐hand panel, Figure 2 in light blue).

The contrasts (SM1) *generate verbs aloud > generate verbs covertly* and (SM2) *listen to non‐words and repeat > listen to non‐words only*, were designed to help isolate activity related to speech production while controlling for the semantic processing/language generation component of the task (present for both conditions in Contrast SM1) and auditory presentation (present in all conditions)—see Figure 2. Both contrasts identified symmetrical bilateral activation in the primary motor cortex and the supplementary motor cortex (see Tables S8 and S9, Supporting Information). Bilateral basal ganglia and insula activity was found in SM2. Within the cerebellum, bilateral activation was observed with Contrast SM2 within Lobule VI close to the midline extending inferiorly into Crus I and in Lobule VIIIa extending superiorly into Lobule VIIb and Crus II. A conjunction of SM1 and SM2 revealed a bilateral pattern of activity that was located exclusively in Lobule VI, near the midline (see right‐hand panel, Figure 2 in yellow).

### 3.4 Activation during VWM

**Figure 3:**
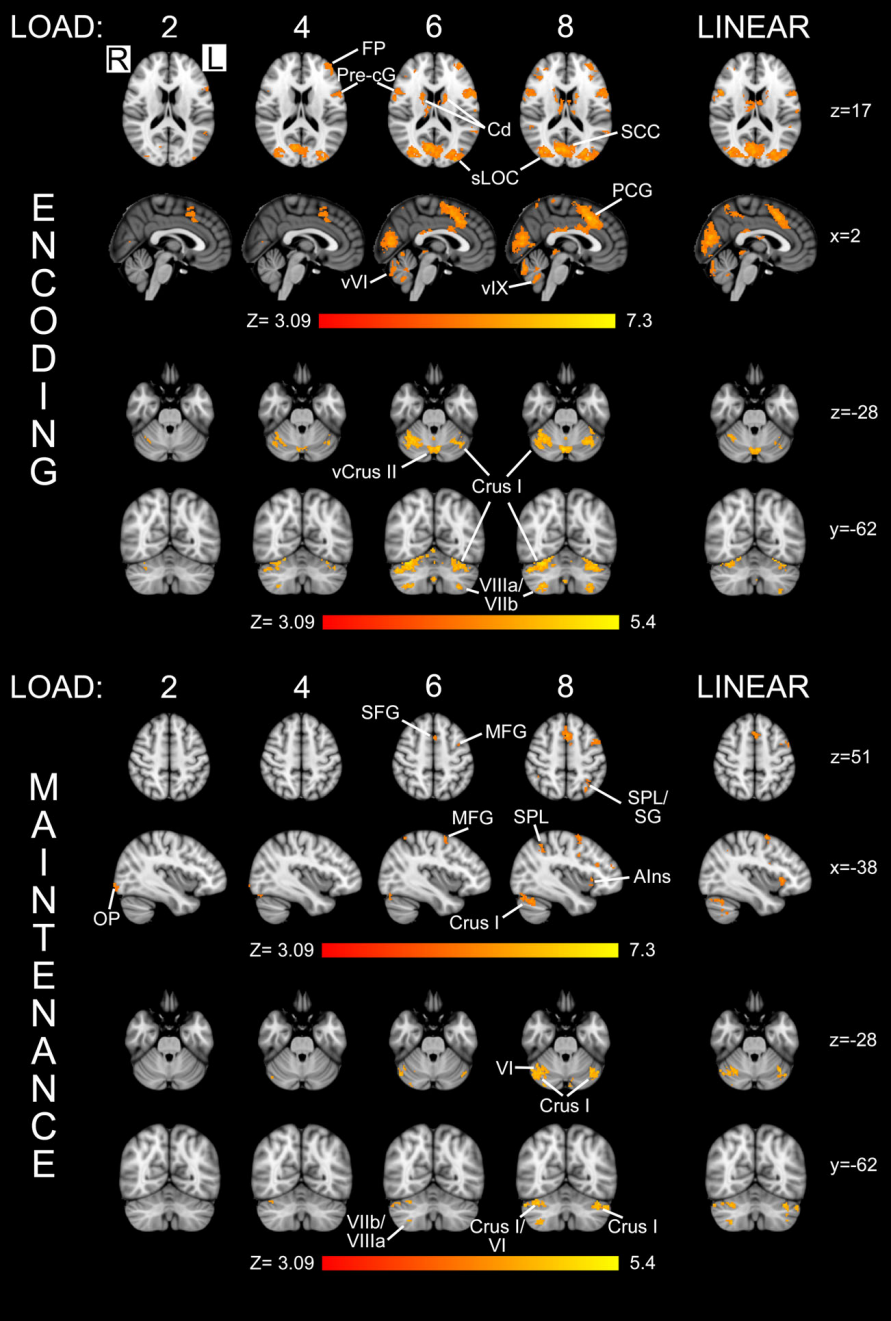
Working memory load and parametric modulation of activity with the cerebellum (*N* = 19). Activity in response to the Sternberg task is shown for two phases of the test: (1) encoding and (2) maintenance for each load (labelled at the top) along with (3) the output of a parametric model of the increasing load (‘LINEAR’). Notably, there was extensive cerebellar activity within the maintenance phase, when subjects were instructed to rehearse (without moving their lips) the letter string previously visible to them. The anatomical locations of the selected slices are given in Montreal Neurological Institute (MNI) coordinates (in mm) on the right of the figure. All activation maps were derived using a cluster forming threshold of *Z* > 3.09 and cluster corrected *p* < .05. AIns, anterior insula; Cd, caudate; FP, frontal pole; MFG, middle frontal gyrus; OP, occipital pole; PCG, paracingulate gyrus; pre‐cG, precentral gyrus; SCC, supracalcarine cortex; SFG, superior frontal gyrus; SG, supramarginal gyrus; sLOC, lateral occipital cortex‐superior division; SPL, superior parietal lobule; vCrus II, vermis Crus II; vVI, Vermis VI; vIX, Vermis IX [Color figure can be viewed at http://wileyonlinelibrary.com]

Activity in response to this event‐related task was modelled independently for the three phases of the Sternberg working memory task (encoding, maintenance, and retrieval) with each phase compared to rest. Results for encoding and maintenance are shown in the top and bottom halves of Figure 3, respectively. For all but the lowest working memory load (two letters), encoding produced widespread activity in the cerebrum including the frontal pole, insular cortex, frontal operculum, precentral gyrus, paracingulate gyrus, and occipital cortex. During maintenance sparse activity was observed, primarily for the highest working memory load, in the middle frontal gyri bilaterally (largest in the left hemisphere), paracingulate gyrus, insular cortex, and frontal operculum. During retrieval (data not shown), which includes a motor response, activity was observed in left precentral and postcentral gyri (button box responses were made with the right hand) and bilaterally in the inferior frontal gyrus and occipital cortex.

Within the cerebellum, during encoding, activity was observed in right Lobule VI, Crus II and VIIb, and Vermis VI. As the working memory load increased, activity in right Lobule VI extended into Crus I, and with the highest load, activity was additionally found in right Lobules VIIb–VIIIa and in Lobules VI and VIIb and Crus II of the left cerebellar hemisphere. The voxel with the highest *Z*‐score in the cerebellum was located in right Lobule VI. During the maintenance phase, there was less extensive activity within Vermis VI, with the voxel of maximum significance located in right Lobule VI, contained within a cluster that extended into Crus I. During retrieval, the largest cluster included the area previously demonstrated to include the finger representation, including Lobules I–IV and Crus I. The voxel with the highest *Z*‐score was, again, located in right Lobule VI. Activity within the posterior lobe was observed bilaterally, in Lobules VIIb and VIIIa on the right and Lobules VIIb and VIIIa, and Crus II on the left. Activation was also found in Vermal VI and VIIIa and Vermis Crus II.

Further analysis was performed to identify areas in the cerebellum where there was a linear relationship between BOLD activity and memory load during the encoding and maintenance phases (right‐hand column of Figure 3). The largest area positively correlated with load during encoding was in Vermis VI and Crus II (see Table S10, Supporting Information). Additional areas which displayed a linear relationship included bilateral areas in Lobules VI and Crus I and right VIIb, VIII, and Crus II. During maintenance, which should contain minimal eye movement as the visual stimulus has been removed from the screen, linearly correlated activity was observed bilaterally in Crus I, extending into Lobule VI on the right and Lobule VI and Crus II on the left (see Table S11, Supporting Information). Small areas in Lobule VIIb were also observed bilaterally. No correlation was found in the vermis.

### 3.5 Frequency map

**Figure 4:**
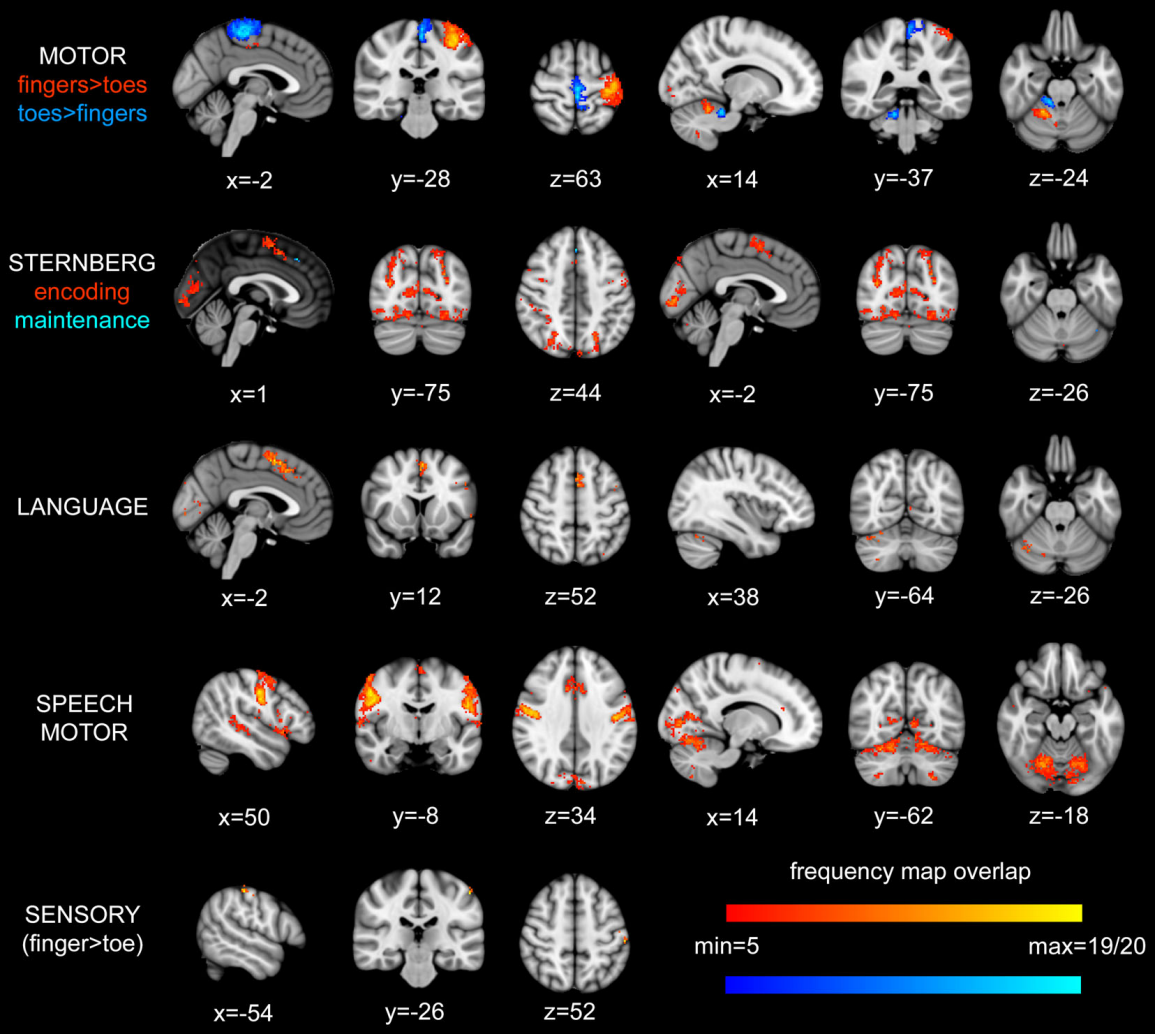
Frequency maps demonstrating the degree of spatial overlap between participants for each paradigm. Statistical maps for each subject were binarised and transformed to standard space where they were added together, a maximum intensity of 19|20 (yellow, light blue) therefore indicated that there was activation at that location in all subjects while an arbitrary minimum of 5 (red, dark blue) out of 19|20 subjects was used for visualisation purposes. For the MOTOR and STERNBERG frequency maps, the two different colours used to represent the degree of overlap in different contrasts within each of these tasks, please see the colour coding below the task name to the left of the images. Note. For cognitive contrasts, only 19 subjects were included in frequency maps, see text for details. For details of location of maximal overlap, see Table S12, Supporting Information [Color figure can be viewed at http://wileyonlinelibrary.com]

To assess the degree of overlap of the applied paradigms between individuals, frequency maps were constructed where maximum intensity of 20 indicates that the same voxel (after transformation to standard space) was activated in all 20 subjects while a minimum intensity of 5 indicates a voxel was activated in only 5 of the 20 subjects (see Figure 4). Note that the maximum intensity for cognitive tasks was 19, due to the exclusion of one subject. The greatest degree of overlap (i.e., consistency) between participants related to somatic tasks, for example, the contrasts fingers > toes and toes > fingers and the speech motor contrast from the language paradigm. While a degree of overlap (yellow colours on frequency maps) was observed for the language task and encoding phase of the working memory task, this was primarily observed within the cerebrum. For tasks not involving explicit motor output (i.e., Sternberg encoding, maintenance, and language), the consistency of spatial activation within the cerebellum was low. For the language contrast, while the degree of consistency was low (i.e., low value on frequency map), the location of greatest overlap was consistent with results from group analysis (cf. Figure 2: Rows 1 and 2 and conjunction) in right Lobule VI and Crus I. The degree of overlap including the location of the voxel(s) of maximal overlap in the cerebrum and cerebellum are shown in Table S12, Supporting Information.

### 3.6 Cerebellar maps

**Figure 5:**
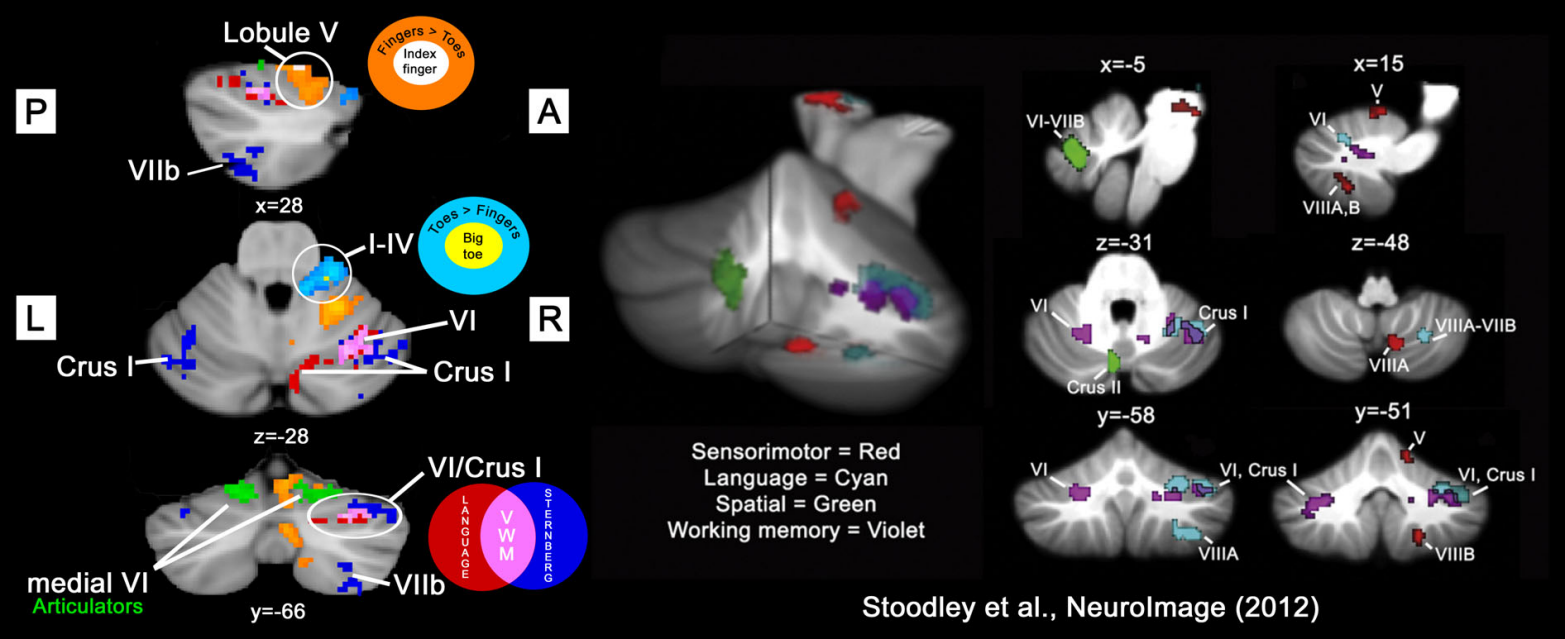
Composite cerebellar map demonstrating topology of sensorimotor and working memory/language function found in this study (on the left). Colour codings for each contrast/conjunction are shown as graphical summaries. For comparison, the results of a previous study (Stoodley et al., 2012) are also shown (on the right). The data obtained in the current study show convergence in both the sensorimotor and cognitive domains. Activity due to passive sensory stimulation of the index finger (white) and big toe (yellow) overlapped with that arising from flexion/extension of the fingers (orange) and toes (light blue), respectively, in the ipsilateral anterior and posterior cerebellar hemisphere. Language (conjunction of language contrasts, red) and Sternberg (linearly increased activity during maintenance of working memory, blue) tasks primarily activated right cerebellar structures. These cognitive tasks also showed a degree of overlap (assessed using a conjunction, pink) in right Crus I/Lobule VI: the putative verbal working memory (VWM) area of the cerebellum. Note that to facilitate visual comparison between studies, data from the current study were flipped horizontally. Statistical maps (including conjunctions) were determined with a cluster forming threshold of *Z* > 3.09 and corrected significance level of *p* < .05, with the exception of contrasts based on vibrotactile stimulation (*p* < .005 uncorrected). (Insert: Stoodley et al., 2012, fig. 1. Reproduced with permission.) [Color figure can be viewed at http://wileyonlinelibrary.com]

A composite map indicating the representation of the different tasks and their anatomical locations within the cerebellum is shown in Figure 5. Movement of the fingers/toes and sensory stimulation of the index finger/big toe on the same body side (right) show overlap within Lobule V (fingers) and Lobules I–IV (toes). Articulatory movement evoked bilateral activity close to the midline in Lobule VI and was adjacent (dorsal in three‐dimensional) to the region representing the fingers. The cognitive paradigms (Sternberg working memory, language task) gave rise to more widespread, sometimes bilateral activity, but again demonstrated a degree of overlap. A linear increase in cerebellar activity in response to increasing working memory load was found primarily within the right cerebellar hemisphere. Activity found within right Lobule VI and Crus I overlapped extensively with the map reflecting activity in response to the language/semantic processing task. A conjunction analysis revealed that the area of overlap was located in Lobule VI/Crus I for these cognitive tasks. For comparison, the results obtained in a similar study (Stoodley et al., 2012) are shown alongside, which reveal broad agreement, particularly overlap between language and working memory (n‐back) tasks in Lobule VI/Crus I. The formal conjunction analysis performed with our data confirms the earlier qualitative observation of overlapping activity within the cognitive domains (Stoodley et al., 2012).

## 4 DISCUSSION

In this study, we mapped, within the same cohort, the spatial localization of sensorimotor, language, and VWM tasks in the cerebellar cortex. By using high‐resolution fMRI and robust acquisition and analysis techniques for improving (a) the accuracy of estimated functional signal and (b) registration to a standard brain atlas, we have determined a map of cerebellar functional localisation. While the acquisition was optimised for visualising cerebellar activation, it also included the whole brain, allowing activation patterns to be compared against expected brain BOLD responses. The findings in healthy subjects demonstrate a clear spatial compartmentalisation of sensorimotor, VWM, speech motor, and language function in the human cerebellum. Applying sensory stimuli, we found an ipsilateral representation in right Lobules I–IV (big toe) and Lobules V and VI/Lobule VIIIa (index finger), which overlapped with the areas activated during movement of the fingers and toes in a matched somatotopic arrangement. The sensorimotor map included mapping speech motor activity, reflecting articulatory movement, which lies adjacent to the hand area in bilateral medial Lobule VI. Higher order cognitive function associated with the language task was estimated via two language contrasts; a conjunction analysis identified Lobule VI–Crus I of the right posterior lobe as the focus of activity. This area coincided with that representing activity during the maintenance phase of the Sternberg task that scaled linearly with increasing working memory load. Localisation was found to be remarkably uniform across individuals for the sensorimotor tests, consistent with an integrative function. By comparison, localisation was generally more variable for the cognitive tests, highlighting the importance of individual cerebellar scans for mapping higher order function.

### 4.1 Sensory and motor tasks

By applying MRI‐compatible piezoelectric tactile stimulators to the index finger and big toe on the right side, we have mapped a passive sensory stimulus of large diameter fibre input to the cerebellum. To control for nonspecific activity related to attention, visual input and eye movement, activity from finger and toe stimulation were subtracted from one another to isolate ‘pure’ sensory components of stimulation. Activity for the finger > toe contrast was observed in the ipsilateral anterior and posterior lobes (Lobules V‐VI and VIIIa/b), whereas activity for the toe > finger contrast was observed in ipsilateral Lobules I–IV only. These findings are in keeping with the known somatotopical arrangement of the cerebellum as detailed in animal electrophysiological studies (Atkins & Apps, 1997; Ekerot & Larson, 1979; Garwicz, Ekerot, & Schouenborg, 1992; Jorntell et al., 2000; Pardoe & Apps, 2002; Pijpers et al., 2006; Snider & Stowell, 1944). However, it should be noted that with the exception of the finger > toe contrast in the cerebrum, the activation maps did not survive correction at the specified statistical threshold. This could be because the cerebellum is involved in discriminating and integrating a combination of sensory inputs, that is, tactile, joint, muscles afferents, meaning that a pure sensory stimulus is not sufficiently salient to produce robust activation. An alternative explanation is that the small and variable activation patterns may be better assessed using multivariate analysis techniques (Wiestler et al., 2011). Consequently, the reported activation within the cerebellum, determined with univariate statistics, can be considered exploratory as determined with an uncorrected threshold of *p* < .005.

During volitional movement of the fingers and toes motor conditions, BOLD activity was subtracted from one another to create contrast images. The fingers > toes (Lobules V/VI and VIIIa/b) and toes > fingers (Lobules I–V and VIIIb–IX) contrasts revealed activity primarily within the ipsilateral right cerebellar hemisphere, consistent with previous fMRI studies (Guell et al., 2018; Schlerf et al., 2010; Spencer et al., 2007; Stoodley et al., 2012). An area of midline activity was observed in oculomotor vermis VI and VIIIa for the contrast fingers > toes. Since activity relating to eye movement was consistent across both tasks, the designed contrasts should have removed any such effect. As a result, we interpret the vermal region of BOLD activity as likely to represent part of the motor map for the upper limb. This interpretation is consistent with previous studies which found an extension of hand and toe areas into the vermis (Grodd et al., 2001; Rijntjes et al., 1999).

Activity from movement of the fingers and toes overlapped with sensory stimulation of the same body part. Evidence for convergent representation of sensory and motor function within the cerebellum has been previously reported in the cat (Eccles, Faber, Murphy, Sabah, & Táboříková, 1971; Eccles, Provini, Strata, & Taborikova, 1968; Snider & Stowell, 1944; Thach Jr., 1967), mice (Proville et al., 2014) and nonhuman primate (Bauswein et al., 1983). However, this organisational principle is not universally accepted (Gao et al., 1996; Hartmann & Bower, 2001; Weeks, Gerloff, Honda, Dalakas, & Hallett, 1999). One other study has investigated cerebellar motor and sensory representation using a similar imaging methodology in humans (Wiestler et al., 2011). Consistent with the present findings, the activation areas in the ipsilateral cerebellum for the sensory (vibrotactile) stimulus overlapped the motor activation areas for both the fingers and toes. Integration of sensorimotor information may enable the cerebellum to form internal models that can predict the sensory consequences of behaviour to fine‐tune task performance (Sokolov, Miall, & Ivry, 2017; Wolpert, Miall, & Kawato, 1998).

### 4.2 Cognitive function

When focusing on higher order cognitive function, we observed activity in cerebellar regions outside the sensorimotor areas located within the anterior and posterior lobes. We adapted a paradigm described by Petersen et al. (1989), who was the first to demonstrate cerebellar involvement in response to a language task (see also Price, 2012; Stoodley et al., 2012). In our study, contrasts designed to isolate the semantic/phonological component of the task identified cerebellar regions located between the two sensorimotor regions bilaterally in Lobule VI, Crus I/Crus II, and Lobules VIIb/VIIIa. Conjunction of two language contrasts revealed a common area of activity located solely within Lobule VI/Crus I, in agreement with other studies (Frings et al., 2006; Guell et al., 2018; Stoodley et al., 2012). While it is possible that speech motor activity might still be present in our language contrast (e.g., verb generation quiet > listen to nouns) due to subvocal articulation during quiet verb generation, we consider this unlikely as even passive listening to speech has been shown to prime brain areas involved in speech production, so should be present in both conditions (Watkins, Strafella, & Paus, 2003). A conjunction of speech motor contrasts (SM1 and SM2) revealed bilateral activity in Lobule VI (adjacent to the hand area). However, activity in right Lobule VIIIa found in SM2 was not reflected in the conjunction but has previously been reported in response to lip movement (Grodd et al., 2001). This illustrates a limitation of using a conservative approach (conjunction analysis) to estimating cerebellar maps.

Disruption of activity within Lobule VI/Crus I using repetitive transcranial magnetic stimulation slows prediction of upcoming sentence content (Lesage, Morgan, Olson, Meyer, & Miall, 2012), while BOLD activity within this region (Crus I/II) increases in proportion to the predictability of sentence outcome, but also in relation to the prediction error between expected and actual sentence outcome (Lesage et al., 2017). Similar to our observation of linearly increasing activity with working memory load in Crus I, Lesage et al. (2017) showed that activity within Crus II increased with working memory demand related to phonological, but not semantic or visuospatial processing. A recent review article by Peterburs, Cheng, and Desmond (2016) summarises the role of cerebellum in performance monitoring. The cerebellum is thought to provide feedforward sensory information to the prefrontal cortex, leading to a creation of an error signal whenever a mismatch occurs between the predicted and actual consequence of an event. This highlights a potential unifying role of the cerebellum, whereby it integrates cognitive and sensory information to provide feedback to higher cortical centres.

Working memory is involved in language processing (Baddeley, 2000; Baddeley, 2003). According to the Baddeley model of working memory (Baddeley, 1992), VWM includes a phonological loop which stores verbal information in a phonological format (i.e., sounds, words, phrases). To maintain phonological information, the phonological loop uses a subvocal rehearsal system for information which would otherwise be lost within seconds (Baddeley, 2003). To assess cerebellar contributions to VWM, we adapted a paradigm (Cairo et al., 2004; Chen & Desmond, 2005a; Chen & Desmond, 2005b; Desmond et al., 1997; Kirschen et al., 2010) based on the Sternberg task (Sternberg, 1966). By focusing on activity during the maintenance phase, where there is minimal visual input and subjects are rehearsing the preceding letter string presented at encoding, and by using direct contrasts between conditions, we sought to isolate VWM activity from other behaviour such as eye movements. This is an important consideration, because previous studies have suggested that cerebellar activation during cognitive tasks may reflect cerebellar involvement in oculomotor control (Doron, Funk, & Glickstein, 2010; Glickstein & Doron, 2008). However, a recent imaging study has shown that performance of the Sternberg task does not result in contamination of cerebellar activity related to eye movements (Peterburs et al., 2016). To further increase the specificity of our findings, we utilised a parametric model to identify cerebellar regions whose activity scaled linearly with increasing VWM load. Load was associated with widespread activity in bilateral Crus I, right Crus II, and right Lobules VI and VIII, with greatest activity in the right cerebellar hemisphere. These results are in broad agreement with others, and suggest extensive areas of the cerebellum are involved in working memory (Chen & Desmond, 2005a; Chen & Desmond, 2005b; Desmond et al., 1997; Guell et al., 2018; Keren‐Happuch et al., 2014; Stoodley et al., 2012; Tomlinson, Davis, Morgan, & Bracewell, 2014). A recent study (Peterburs, Blevins, Sheu, & Desmond, 2019), which also used a Sternberg paradigm, found that BOLD activity in Lobule VIII (previously shown to be involved in maintenance, Kirschen et al., 2010), increased with working memory load. Taken together, these findings provide evidence of a role for the cerebellum in sequence rehearsal, detection, and prediction in relation to VWM.

One important difference concerns comparison of our findings with the work of Stoodley et al., 2012. Although they found spatial overlap in the cerebellum between a language task: verb generation (right Lobule VI‐Crus I, extending into VIIIA) and working memory using the n‐back task (bilateral activation of Lobules VI and VII), their conjunction analysis did not confirm this observation. In the present study, conjunction of linear working memory load (i.e., during maintenance, see Figure 3) with the result from a conjunction of two independent language contrasts (see Figure 2) identified an area within right Lobule VI/Crus I (see Figure 5), which we believe forms a locus of activity involved in VWM within the cerebellum. Several factors may explain the difference between Stoodley et al. (2012) and the current results (see Figure 5). Our investigation has greater power to detect an effect (*N* = 20 subjects vs. *N* = 9 in their study), and the additional steps we took to account for sources of physiological noise increase confidence in the findings. Additionally, the working memory load of a two‐back task used by Stoodley et al. (2012) may not be sufficiently cognitively demanding to produce substantial activity in the cerebellum.

A recent landmark study, utilised a large dataset of 787 subjects from the HCP to evaluate the correspondence between resting‐state and task‐based cerebellar activity across motor and cognitive domains (Guell et al., 2018). Activity from n‐back and language tasks did not overlap, which is surprising given that both rely on working memory (Baddeley & Hitch, 1974), and contradicts earlier findings from the same group (Stoodley et al., 2012) and results from the present study. Possible explanations for this may relate to the HCP's choice of the n‐back task to examine working memory, which shows poor construct validity when compared to conventional measures (Jaeggi, Buschkuehl, Perrig, & Meier, 2010; Jarrold & Towse, 2006; Kane, Conway, Miura, & Colflesh, 2007; Miller, Price, Okun, Montijo, & Bowers, 2009). Furthermore, the n‐back task employed a nonstandard design: simultaneously examining category specific representations and working memory, using pictures of places, faces, tools and body parts. These stimuli reliably engage distinct cortical regions (Barch, et al., 2013), and thus may have also produced distinct cerebellar activity. Here, we chose a working memory paradigm that explicitly addressed encoding and maintenance of information (Baddeley, 2003) and revealed parametric activity that increased with working memory load. By comparison, the HCP's language task (Binder et al., 2011) compared activity during a language comprehension task with that due to mental arithmetic, which appear to be poorly matched given that language is involved in both tasks. The language task used in the present study (Petersen et al., 1989) was chosen to control for motor and nonmotor components, and provided two separate estimates of language‐related activity that were then combined through a conjunction analysis. We believe our approach of using carefully controlled tasks and a conservative approach to determining maps representative of language and working memory function allows for a confident claim that they share a common underlying neural substrate in the cerebellum—with the most parsimonious explanation for this overlap being that they both rely on working memory.

### 4.3 Intersubject variability

Mixed effects group fMRI analysis maps the average population response (Mumford & Poldrack, 2007), which will obscure any individual differences in the cohort (Seghier & Price, 2016). To overcome this limitation, we created frequency maps of intersubject variability in cerebellar activation patterns to assess the degree of consistency between subjects for each task. The motor paradigms revealed BOLD activity that was remarkably uniform in localisation between participants. However, vibrotactile (sensory) stimulation produced almost no overlapping voxels between subjects. Rather than overinterpret this null result, we suggest that the inherently weaker activity induced by vibrotactile stimulation may have led to this finding. Similarly, localisation was much more variable for the cognitive tests. The variability could be due to several possibilities that are not mutually exclusive. These include differences between individuals in the strategies they use to perform cognitive tasks, which in turn may be dependent on other factors (e.g., motivation, genetic, intrinsic ability at the tasks). We cannot, however, exclude the possibility that cognitive tasks are less potent in generating BOLD activity in the cerebellum. In terms of anatomical consistency, we note that the sensorimotor tasks (hand/foot movement) were associated with rather small areas of activity; however, these smaller patches were clearly aligned between subjects, whereas the more diffuse activity associated with cognitive tasks did not. Of note, is a recent study by Marek et al. (2018) who showed that individual variability in cognitive networks is greater than in motor networks measured from resting‐state functional connectivity data. Therefore, the cognitive variability between individuals may reflect genuine individual differences in anatomical representation of higher order cognitive function within the cerebellum, which may emerge during development (Moore, D'Mello, McGrath, & Stoodley, 2017).

Whatever the underlying reasons for the intersubject variability, the present results suggest that individual scans are necessary if fMRI were to be used clinically—especially when attempting to understanding how individual variability can lead to differences in clinical outcomes. For example, posterior fossa surgery can lead to cerebellar mutism syndrome—a transient loss of speech output, subsequently associated with an impairment of fluency, articulation, and modulation of speech, and is a recognised complication that develops in one in three children (Pitsika & Tsitouras, 2013; Wells et al., 2010) following surgery for cerebellar or fourth ventricular tumours (Rekate, Grubb, Aram, Hahn, & Ratcheson, 1985). Our maps of intersubject variability in cognitive function may therefore be of direct relevance when assessing why some individuals develop cerebellar mutism and others do not. Individualised mapping of the cerebellum may also help to understand the range of cognitive deficits observed in cerebellar clinical populations (Schmahmann & Sherman, 1998).

In summary, the results from our study show a clear spatial compartmentalisation of sensorimotor and cognitive functions within the human cerebellar cortex (summarised in Figure 5). Previous resting‐state fMRI studies (Buckner, Krienen, Castellanos, Diaz, & Yeo, 2011; O'Reilly, Beckmann, Tomassini, Ramnani, & Johansen‐Berg, 2010) have identified a parcellation of the cerebellum. In particular, the study by Buckner et al. (2011) demonstrated involvement of Lobule VI in both sensorimotor and cognitive domains, in partial support of the findings from the current study. By using task‐based fMRI, we demonstrate a convergence of sensory input and motor output within the sensorimotor map and a similar convergence of cognitive functions involved in VWM and language in the cognitive map. However, our frequency map results emphasise that caution must be used when extending group fMRI cerebellar results to the individual, particularly in the cognitive domain.

## CONFLICT OF INTEREST

The authors declare no potential conflict of interest.

## Supporting information


**Table S1** Participant neuropsychology assessment results. Individual results, mean and *SD* are shown for five neuropsychological assessments.
**Table S2** Detailed description of clusters of activation for finger greater than toe contrast of the motor paradigm, showing the activation cluster size, maximum intensity, coordinates of voxel with maximum intensity for clusters. Anatomical localisation based on overlap between the *cluster* and cortical/subcortical/ cerebellar probabilistic atlases present in FSL. Results from mixed effects modelling were obtained using a cluster forming threshold of Z > 3.09 and corrected *p* < 0.05. †Obtained using a cerebellar mask (see Methods).
**Table S3** Detailed description of clusters of activation for toe greater than finger contrast of the motor paradigm, showing the activation cluster size, maximum intensity, coordinates of voxel with maximum intensity for clusters. Anatomical localisation based on overlap between the *cluster* and cortical/subcortical/cerebellar probabilistic atlases present in FSL. Results from mixed effects modelling were obtained using a cluster forming threshold of Z > 3.09 and corrected *p* < 0.05. †Obtained using a cerebellar mask (see Methods).
**Table S4** Detailed description of clusters of activation for finger greater than toe contrast of the vibrotactile paradigm, showing the activation cluster size, maximum intensity, coordinates of voxel with maximum intensity for clusters. Anatomical localisation based on overlap between the *cluster* and cortical/subcortical/cerebellar probabilistic atlases present in FSL. Results from mixed effects modelling were obtained (uncorrected, p < 0.005). †Obtained using a cerebellar mask (see Methods).
**Table S5** Detailed description of clusters of activation for toe greater than finger contrast of the vibrotactile paradigm showing the activation cluster size, maximum intensity, coordinates of voxel with maximum intensity for clusters. Anatomical localisation based on overlap between the *cluster* and cortical/subcortical/cerebellar probabilistic atlases present in FSL. Results from mixed effects modelling were obtained (uncorrected, p < 0.005). †Obtained using a cerebellar mask (see Methods).
**Table S6** Detailed description of clusters of activation for the language 1 (L1) paradigm (generate verbs covertly‐listen to nouns only) showing the activation cluster size, maximum intensity, coordinates of voxel with maximum intensity for clusters. Anatomical localisation based on overlap between the cluster and cortical/subcortical/cerebellar probabilistic atlases present in FSL. †Obtained using a cerebellar mask (see Methods). Results from mixed effects modelling were obtained using a cluster forming threshold of Z > 3.09 and corrected *p* < 0.05. †Obtained using a cerebellar mask (see Methods).
**Table S7**: Detailed description of clusters of activation for the language 2 (L2) paradigm (generate verbs aloud‐listen to non‐words and repeat) showing the activation cluster size, maximum intensity, coordinates of voxel with maximum intensity for clusters. Anatomical localisation based on overlap between the cluster and cortical/subcortical/cerebellar probabilistic atlases present in FSL. †Obtained using a cerebellar mask (see Methods). Results from mixed effects modelling were obtained using a cluster forming threshold of Z > 3.09 and corrected *p* < 0.05. †Obtained using a cerebellar mask (see Methods).
**Table S8**: Detailed description of clusters of activation for the Speech Motor 1 (SM1) paradigm (generate verbs aloud‐generate verbs covertly) showing the activation cluster size, maximum intensity, coordinates of voxel with maximum intensity for clusters. Anatomical localisation based on overlap between the *cluster* and cortical/subcortical/cerebellar probabilistic atlases present in FSL. †Obtained using a cerebellar mask (see Methods). Results from mixed effects modelling were obtained using a cluster forming threshold of Z > 3.09 and corrected *p* < 0.05. †Obtained using a cerebellar mask (see Methods).
**Table S9** Detailed description of clusters of activation for the Speech Motor 2 (SM2) paradigm (listen non words and repeat‐listen to nouns only) showing the activation cluster size, maximum intensity, coordinates of voxel with maximum intensity for clusters. Anatomical localisation based on overlap between the *cluster* and cortical/subcortical/cerebellar probabilistic atlases present in FSL. †Obtained using a cerebellar mask (see Methods). Results from mixed effects modelling were obtained using a cluster forming threshold of Z > 3.09 and corrected *p* < 0.05. †Obtained using a cerebellar mask (see Methods).
**Table S10** Detailed description of clusters of activation for the verbal working memory, linear encoding paradigm showing the activation cluster size, maximum intensity, coordinates of voxel with maximum intensity for clusters. Anatomical localisation based on overlap between the cluster and cortical/subcortical/cerebellar probabilistic atlases present in FSL. †Obtained using a cerebellar mask (see Methods). Results from mixed effects modelling were obtained using a cluster forming threshold of Z > 3.09 and corrected *p* < 0.05.
**Table S11** Detailed description of clusters of activation for the showing the verbal working memory, linear maintenance paradigm showing the activation cluster size, maximum intensity, coordinates of voxel with maximum intensity for clusters. Anatomical localisation based on overlap between the cluster and cortical/subcortical/cerebellar probabilistic atlases present in FSL. †Obtained using a cerebellar mask (see Methods). Results from mixed effects modelling were obtained using a cluster forming threshold of Z > 3.09 and corrected *p* < 0.05.
**Table S12** Summary of maximal overlap frequency and anatomical locations for frequency maps. The number in brackets under locations columns is the corresponding frequency for the reported maximal voxel. Note: the input data used when generating frequency maps for the Sternberg task was based on the parametric model during the maintenance phase

**Figure S1** Five language contrasts (versus rest). In response to the separate portions of the language task activity was seen across cortical (predominantly left sided, including Broca's and Wernicke's) and sub‐cortical regions in the cerebrum and in the cerebellum (predominantly right sided). Labelled activity is as follows: HG, Heschl's gyrus; PCG, paracingulate gyrus; CGpre, precentral gyrus; CGpo, postcentral gyrus; IFGpo, inferior frontal gyrus, pars opercularis; IFGpt, inferior frontal gyrus, pars triangularis; STGpd, superior temporal gyrus, posterior division; PT, planum temporale. Activation was determined using a cluster forming threshold of Z > 3.09 and cluster corrected *p* < 0.05 for the whole brain (left hand side) and for the cerebellum alone (right hand side) using a mask, see Methods.

## Data Availability

The data that support the findings of this study are available from the corresponding author upon reasonable request.
