# Supplementary material for: Sensorimotor, language, and working memory representation within the human cerebellum
Source: Hum Brain Mapp. 2019 Jul 30;40(16):4732–47. doi: 10.1002/hbm.24733 (PMC6865458; doi:10.1002/hbm.24733)
Supplement: Supplementary file 1 — Table S1 Participant neuropsychology assessment results. Individual results, mean and SD are shown for five neuropsychological assessments. Table S2 Detailed description of clusters of activation for finger greater than toe contrast of the motor paradigm, showing the activation cluster size, maximum intensity, coordinates of voxel with maximum intensity for clusters. Anatomical localisation based on overlap between the cluster and cortical/subcortical/ cerebellar probabilistic atlases present in FSL. Results from mixed effects modelling were obtained using a cluster forming threshold of Z > 3.09 and corrected p < 0.05. †Obtained using a cerebellar mask (see Methods). Table S3 Detailed description of clusters of activation for toe greater than finger contrast of the motor paradigm, showing the activation cluster size, maximum intensity, coordinates of voxel with maximum intensity for clusters. Anatomical localisation based on overlap between the cluster and cortical/subcortical/cerebellar probabilistic atlases present in FSL. Results from mixed effects modelling were obtained using a cluster forming threshold of Z > 3.09 and corrected p < 0.05. †Obtained using a cerebellar mask (see Methods). Table S4 Detailed description of clusters of activation for finger greater than toe contrast of the vibrotactile paradigm, showing the activation cluster size, maximum intensity, coordinates of voxel with maximum intensity for clusters. Anatomical localisation based on overlap between the cluster and cortical/subcortical/cerebellar probabilistic atlases present in FSL. Results from mixed effects modelling were obtained (uncorrected, p < 0.005). †Obtained using a cerebellar mask (see Methods). Table S5 Detailed description of clusters of activation for toe greater than finger contrast of the vibrotactile paradigm showing the activation cluster size, maximum intensity, coordinates of voxel with maximum intensity for clusters. Anatomical localisation based on overlap between the cl [file HBM-40-4732-s001.docx]

**Supplementary Tables**

Ashida, Cerminara, Edwards, Apps, Brooks

**Supplementary Table 1** Participant neuropsychology assessment results. Individual results, mean and SD are shown for five neuropsychological assessments.

| Subject | Age | Sex | Handedness  score | Digit span  SSA | Arithmetic  SSA | Letter number  sequencing SSA | Comprehension  SSA | Pegboard time (number dropped) |
| --- | --- | --- | --- | --- | --- | --- | --- | --- |
| 1 | 33 | F | 1 | 11 | 14 | 8 | 12 | 58 (0) |
| 2 | 44 | F | 1 | 13 | 14 | 6 | 12 | 57 (0) |
| 3 | 26 | F | 1 | 10 | 9 | 6 | 9 | 62 (0) |
| 4 | 26 | M | 0.6 | 10 | 12 | 6 | 15 | 70 (1) |
| 5 | 29 | M | 1 | 8 | 11 | 5 | 11 | 62 (0) |
| 6 | 23 | F | 0.8 | 15 | 15 | 7 | 10 | 60 (0) |
| 7 | 27 | F | 1 | 11 | 11 | 8 | 14 | 64 (0) |
| 8 | 29 | F | 1 | 10 | 14 | 6 | 11 | 65 (0) |
| 9 | 25 | F | 1 | 5 | 11 | 5 | 10 | 61 (0) |
| 10 | 28 | M | 1 | 9 | 11 | 5 | 13 | 64 (0) |
| 11 | 24 | F | 1 | 13 | 12 | 6 | 14 | 57 (0) |
| 12 | 42 | F | 1 | 13 | 13 | 7 | 16 | 72 (1) |
| 13 | 23 | F | 1 | 7 | 15 | 6 | 13 | 52 (0) |
| 14 | 35 | M | 1 | 12 | 13 | 7 | 9 | 69 (0) |
| 15 | 24 | M | 1 | 8 | 19 | 6 | 13 | 101 (0) |
| 16 | 31 | F | 1 | 10 | 8 | 7 | 12 | 53 (0) |
| 17 | 26 | M | 1 | 10 | 14 | 6 | 11 | 64 (0) |
| 18 | 31 | F | 1 | 12 | 15 | 7 | 9 | 58 (0) |
| 19 | 27 | F | 1 | 8 | 11 | 6 | 10 | 60 (0) |
| 20 | 32 | F | 1 | 11 | 10 | 6 | 12 | 53 (0) |
| Mean | 29.3 |  |  | 10.3 | 12.6 | 6.3 | 11.8 | 63.6 |
| SD | 5.78 |  |  | 2.39 | 2.52 | 0.86 | 2.02 | 10.2 |
| Normative Mean |  |  |  | 10 | 10 | 10 | 10 | 65.13 |
| Normative SD |  |  |  | 3 | 3 | 3 | 3 | 9.16 |
| *Z score |  |  |  | 0.45 | 3.88 | -5.52 | 2.68 | -0.74 |
| P value |  |  |  | p>0.3 | p<0.0001 | p<0.0001 | p<0.005 | p>0.2 |

SSA: scaled score by age, SD: standard deviation. *Z scores were calculated using the mean and SD from the normative data from WAIS-IV Administration and Scoring Manual, Lafayette Grooved Pegboard user instructions.

**Supplementary Table 2** Detailed description of clusters of activation for finger greater than toe contrast of the motor paradigm ) showing the activation cluster size, maximum intensity, coordinates of voxel with maximum intensity for clusters. Anatomical localisation based on overlap between the *cluster* and cortical/subcortical/ cerebellar probabilistic atlases present in FSL. Results from mixed effects modelling were obtained using a cluster forming threshold of Z>3.09 and corrected P<0.05. †Obtained using a cerebellar mask (see Methods).

| **Voxels** | **Peak intensity max voxel** | **Max X** | **Max Y** | **Max Z** | **Anatomical location of cluster** |
| --- | --- | --- | --- | --- | --- |
| 1505 | 6.58 | -40 | -24 | 54 | Precentral Gyrus (17.6%) Postcentral Gyrus (30.9%) |
| 175 | 4.69 | -12 | -92 | -6 | Lingual Gyrus (17.3%); Occipital Fusiform Gyrus (24.8%); Occipital Pole (7.1%) |
| 75 | 4.78 | -8 | -14 | 48 | Precentral Gyrus (31.2%); Juxtapositional Lobule Cortex (formerly Supplementary Motor Cortex) (11.5%); Cingulate Gyrus, anterior division (5.0%); Cingulate Gyrus, posterior division (23.5%) |
| 70 | 4.32 | 16 | -86 | -4 | Lingual Gyrus (17.1%); Occipital Fusiform Gyrus (22.8%); Occipital Pole (12.3%) |
| 28 | 3.8 | 46 | -24 | 42 | Postcentral Gyrus (49.7%); Supramarginal Gyrus, anterior division (12.2%) |
| 713† | 6.66 | 20 | -48 | -24 | Right V (43.4%); Vermis VI (5.1%); Right VI (33.3%) |
| 182† | 5.83 | 14 | -62 | -48 | Vermis VIIIa (8.2%); Right VIIIa (36.2%); Right VIIIb (34.8%); |

**Supplementary Table 3** Detailed description of clusters of activation for toe greater than finger contrast of the motor paradigm, showing the activation cluster size, maximum intensity, coordinates of voxel with maximum intensity for clusters. Anatomical localisation based on overlap between the *cluster* and cortical/subcortical/cerebellar probabilistic atlases present in FSL. Results from mixed effects modelling were obtained using a cluster forming threshold of Z>3.09 and corrected P<0.05. †Obtained using a cerebellar mask (see Methods).

| **Voxels** | **Peak intensity max voxel** | **Max X** | **Max Y** | **Max Z** | **Anatomical location of cluster** |
| --- | --- | --- | --- | --- | --- |
| 723 | 6.76 | -2 | -26 | 68 | Precentral Gyrus (31.6%); Postcentral Gyrus (13.1%); Juxtapositional Lobule Cortex (formerly Supplementary Motor Cortex) (5.3%) |
| 383 | 6.74 | 14 | -38 | -24 | Brain-Stem (14.1%) |
| 44 | 4.17 | 8 | -6 | 44 | Juxtapositional Lobule Cortex (formerly Supplementary Motor Cortex) (26.3%); Cingulate Gyrus, anterior division (35.6%); Cingulate Gyrus, posterior division (7.1%) |
| 24 | 5.08 | -34 | -22 | 16 | Insular Cortex (38.7%); Central Opercular Cortex (6.4%); Parietal Operculum Cortex (5.2%) |
| 22 | 4.07 | -32 | -8 | 6 | Left Putamen (53.9%) |
| 723 | 6.76 | -2 | -26 | 68 | Precentral Gyrus (31.6%); Postcentral Gyrus (13.1%); Juxtapositional Lobule Cortex (formerly Supplementary Motor Cortex) (5.3%) |
| 372† | 6.74 | 14 | -38 | -24 | Right I-IV (61.8%); Right V (11.1%) |
| 17† | 3.6 | 18 | -48 | -56 | Right VIIIb (80.2%); Right IX (15.8%) |

**Supplementary Table 4** Detailed description of clusters of activation for finger greater than toe contrast of the vibrotactile paradigm ) showing the activation cluster size, maximum intensity, coordinates of voxel with maximum intensity for clusters. Anatomical localisation based on overlap between the *cluster* and cortical/subcortical/cerebellar probabilistic atlases present in FSL. Results from mixed effects modelling were obtained (uncorrected, p<0.005). †Obtained using a cerebellar mask (see Methods).

| **Voxels** | **Peak intensity max voxel** | **Max X** | **Max Y** | **Max Z** | **Anatomical location of cluster** |
| --- | --- | --- | --- | --- | --- |
| 428 | 4.69 | -46 | -16 | 58 | Precentral Gyrus (8.9%); Postcentral Gyrus (46.9%) |
| 12 | 2.81 | -48 | -22 | 20 | Central Opercular Cortex (45.3%); Parietal Operculum Cortex (29.2%) |
| 10 | 3.32 | -54 | -18 | 12 | Central Opercular Cortex (27.0%); Heschl's Gyrus (includes H1 and H2) (35.3%); Planum Temporale (10.6%) |
| 8† | 2.97 | 30 | -50 | -22 | Right V (10.6%); Right VI (65.1%) |
| 5† | 2.9 | 16 | -62 | -52 | Right VIIIa (63.6%); Right VIIIb (35.6%) |

**Supplementary Table 5** Detailed description of clusters of activation for toe greater than finger contrast of the vibrotactile paradigm showing the activation cluster size, maximum intensity, coordinates of voxel with maximum intensity for clusters. Anatomical localisation based on overlap between the *cluster* and cortical/subcortical/cerebellar probabilistic atlases present in FSL. Results from mixed effects modelling were obtained (uncorrected, p<0.005). †Obtained using a cerebellar mask (see Methods).

| **Voxels** | **Peak intensity max voxel** | **Max X** | **Max Y** | **Max Z** | **Anatomical location of cluster** |
| --- | --- | --- | --- | --- | --- |
| 7 | 3.28 | -2 | -30 | 66 | Precentral Gyrus (51.6%); Postcentral Gyrus (11.0%) |
| 7 | 3.07 | -6 | -20 | 64 | Precentral Gyrus (53.6%); Juxtapositional Lobule Cortex (formerly Supplementary Motor Cortex) |
| 6 | 3.4 | -34 | -24 | 16 | Insular Cortex (37.7%); Central Opercular Cortex (7.7%); Parietal Operculum Cortex (9.2%) |
|  |  |  |  |  |  |
| 2† | 2.67 | 20 | -36 | -26 | Right I-IV (65.0%); Right V (21.5%) |
| 1† | 3.09 | 22 | -30 | -26 | Right I-IV (62.0%); Right V (6.0%) |

**Supplementary Table 6** Detailed description of clusters of activation for the language 1 (L1) paradigm (generate verbs covertly-listen to nouns only) showing the activation cluster size, maximum intensity, coordinates of voxel with maximum intensity for clusters. Anatomical localisation based on overlap between the cluster and cortical/subcortical/cerebellar probabilistic atlases present in FSL. †Obtained using a cerebellar mask (see Methods). Results from mixed effects modelling were obtained using a cluster forming threshold of Z>3.09 and corrected P<0.05. †Obtained using a cerebellar mask (see Methods).

| **Voxels** | **Peak intensity max voxel** | **Max X** | **Max Y** | **Max Z** | **Anatomical location of cluster** |
| --- | --- | --- | --- | --- | --- |
| 1569 | 5.4 | -12 | -82 | -10 | Intracalcarine Cortex (10.0%); Lingual Gyrus (12.4%); Occipital Fusiform Gyrus (5.5%) |
| 1056 | 4.66 | -42 | 14 | 30 | Insular Cortex (5.9%); Middle Frontal Gyrus (14.2%); Inferior Frontal Gyrus, pars triangularis (5.4%); Inferior Frontal Gyrus, pars opercularis (15.4%); Precentral Gyrus (6.7%); Frontal Orbital Cortex (5.4%); Frontal Operculum Cortex (5.2%) |
| 1004 | 4.82 | -4 | 10 | 56 | Superior Frontal Gyrus (10.8%); Juxtapositional Lobule Cortex (formerly Supplementary Motor Cortex) (11.3%); Paracingulate Gyrus (31.5%); Cingulate Gyrus, anterior division (11.4%) |
| 95 | 4.39 | 34 | 26 | 2 | Insular Cortex (30.0%); Frontal Orbital Cortex (21.8%); Frontal Operculum Cortex (12.3%) |
| 55 | 4.01 | -28 | -66 | 48 | Lateral Occipital Cortex, superior division (58.0%) |
| 54 | 4.37 | -60 | -38 | 6 | Superior Temporal Gyrus, posterior division (42.3%); Middle Temporal Gyrus, posterior division (17.7%); Middle Temporal Gyrus, temporooccipital part (6.2%); Supramarginal Gyrus, posterior division (5.6%) |
| 30 | 3.83 | -32 | -2 | 64 | Superior Frontal Gyrus (6.3%); Middle Frontal Gyrus (34.6%); Precentral Gyrus (12.8%) |
| 634† | 4.99 | 8 | -76 | -18 | Right VI (45.2%); Right Crus I (39.8%); Right Crus II (7.3%) |
| 88† | 4.2 | -38 | -62 | -26 | Left VI (43.4%); Left Crus I (55.6%) |
| 56† | 4.06 | 32 | -64 | -48 | Right Crus II (9.2%); Right VIIb (47.3%); Right VIIIa (26.4%) |
| 19† | 3.63 | -8 | -76 | -18 | Left VI (77.1%); Vermis VI (18.9%) |

**Supplementary Table 7**: Detailed description of clusters of activation for the language 2 (L2) paradigm (generate verbs aloud-listen to non-words and repeat) showing the activation cluster size, maximum intensity, coordinates of voxel with maximum intensity for clusters. Anatomical localisation based on overlap between the cluster and cortical/subcortical/cerebellar probabilistic atlases present in FSL. †Obtained using a cerebellar mask (see Methods). Results from mixed effects modelling were obtained using a cluster forming threshold of Z>3.09 and corrected P<0.05. †Obtained using a cerebellar mask (see Methods).

| **Voxels** | **Peak intensity max voxel** | **Max X** | **Max Y** | **Max Z** | **Anatomical location of cluster** |
| --- | --- | --- | --- | --- | --- |
| 278 | 4.93 | -34 | -62 | 48 | Superior Parietal Lobule (9.1%); Angular Gyrus (5.6%); Lateral Occipital Cortex, superior division (45.0%) |
| 255 | 4.52 | -42 | 6 | 50 | Superior Frontal Gyrus (10.7%); Middle Frontal Gyrus (32.1%); Precentral Gyrus (7.2%) |
| 173 | 4.49 | 0 | 18 | 44 | Superior Frontal Gyrus (12.0%); Paracingulate Gyrus (52.7%); Cingulate Gyrus, anterior division (7.1%) |
| 65 | 4.28 | -46 | 22 | 26 | Middle Frontal Gyrus (25.0%); Inferior Frontal Gyrus, pars triangularis (14.5%); Inferior Frontal Gyrus, pars opercularis (8.9%) |
| 59 | 4.4 | 4 | -82 | -14 | Lingual Gyrus (40.2%); Occipital Fusiform Gyrus (6.2%) |
| 51 | 4.47 | -58 | -50 | -6 | Middle Temporal Gyrus, posterior division (7.6%); Middle Temporal Gyrus, temporooccipital part (39.6%) |
| 39 | 4.19 | -22 | 18 | 44 | Superior Frontal Gyrus (23.9%); Middle Frontal Gyrus (8.5%) |
| 958† | 5.33 | 42 | -66 | -56 | Right VI (14.2%); Right Crus I (26.7%); Right Crus II (38.2%); Right VIIb (8.2%) |
| 81† | 4.11 | -34 | -50 | -58 | Left VIIb (12.0%); Left VIIIa (48.3%); Left VIIIb (27.5%) |
| 56† | 4.57 | 30 | -54 | -58 | Right VIIb (12.2%); Right VIIIa (55.5%); Right VIIIb (15.3%) |
| 28† | 3.69 | -30 | -58 | -34 | Left VI (22.2%); Left Crus I (69.5%) |
| 19† | 3.73 | 32 | -44 | -52 | Right VIIb (10.2%); Right VIIIa (69.5%) |

**Supplementary Table 8**: Detailed description of clusters of activation for the Speech Motor 1 (SM1) paradigm (generate verbs aloud-generate verbs covertly) showing the activation cluster size, maximum intensity, coordinates of voxel with maximum intensity for clusters. Anatomical localisation based on overlap between the *cluster* and cortical/subcortical/cerebellar probabilistic atlases present in FSL. †Obtained using a cerebellar mask (see Methods). Results from mixed effects modelling were obtained using a cluster forming threshold of Z>3.09 and corrected P<0.05. †Obtained using a cerebellar mask (see Methods).

| **Voxels** | **Peak intensity max voxel** | **Max X** | **Max Y** | **Max Z** | **Anatomical location of cluster** |
| --- | --- | --- | --- | --- | --- |
| 440 | 5.36 | 50 | -12 | 38 | Precentral Gyrus (29.5%); Postcentral Gyrus (26.2%) |
| 381 | 5.31 | -52 | -12 | 40 | Precentral Gyrus (28.1%); Postcentral Gyrus (29.1%) |
| 113 | 4.6 | -2 | -42 | -54 | Brain-Stem (83.6%) |
| 84 | 4.45 | -4 | -82 | 40 | Lateral Occipital Cortex, superior division (5.7%); Precuneous Cortex (17.4%); Cuneal Cortex (34.5%) |
| 24 | 3.85 | 4 | 32 | 20 | Paracingulate Gyrus (16.7%); Cingulate Gyrus, anterior division (62.2%) |
| 242† | 5.2 | 16 | -62 | -18 | Right V (19.8%); Right VI (72.3%) |
| 215† | 5.03 | -12 | -60 | -22 | Left V (10.6%); Left VI (81.5%) |
| 120† | 4.71 | -34 | -52 | -54 | Left VIIb (10.6%); Left VIIIa (54.0%); Left VIIIb (27.7%) |
| 30† | 4.15 | -26 | -60 | -58 | Left VIIb (16.8%); Left VIIIa (68.8%); Left VIIIb (12.0%) |
| 22† | 3.96 | 36 | -52 | -50 | Right Crus II (7.0%); Right VIIb (43.7%); Right VIIIa (43.7%) |
| 20† | 3.97 | -22 | -76 | -36 | Left Crus I (27.4%); Left Crus II (68.2%) |

**Supplementary Table 9** Detailed description of clusters of activation for the Speech Motor 2 (SM2) paradigm (listen non words and repeat-listen to nouns only) showing the activation cluster size, maximum intensity, coordinates of voxel with maximum intensity for clusters. Anatomical localisation based on overlap between the *cluster* and cortical/subcortical/cerebellar probabilistic atlases present in FSL. †Obtained using a cerebellar mask (see Methods). Results from mixed effects modelling were obtained using a cluster forming threshold of Z>3.09 and corrected P<0.05. †Obtained using a cerebellar mask (see Methods).

| **Voxels** | **Peak intensity max voxel** | **Max X** | **Max Y** | **Max Z** | **Anatomical location of cluster** |
| --- | --- | --- | --- | --- | --- |
| 4786 | 6.03 | 20 | -64 | -18 | Intracalcarine Cortex (13.8%); Cuneal Cortex (5.7%); Lingual Gyrus (14.0%) |
| 1849 | 5.85 | 56 | -4 | 34 | Precentral Gyrus (17.3%); Superior Temporal Gyrus, posterior division (6.7%); Postcentral Gyrus (9.0%) |
| 1500 | 5.95 | -46 | -14 | 38 | Precentral Gyrus (27.3%); Postcentral Gyrus (15.7%) |
| 1288 | 5.27 | -4 | -2 | 60 | Superior Frontal Gyrus (5.5%); Juxtapositional Lobule Cortex (formerly Supplementary Motor Cortex) (15.8%); Paracingulate Gyrus (18.2%); Cingulate Gyrus, anterior division (22.9%) |
| 244 | 4.4 | -34 | 20 | 12 | Insular Cortex (18.7%); Frontal Orbital Cortex (13.0%); Frontal Operculum Cortex (16.3%) |
| 186 | 4.62 | -38 | -32 | 14 | Supramarginal Gyrus, posterior division (12.3%); Parietal Operculum Cortex (30.4%); Planum Temporale (21.5%) |
| 129 | 4.39 | -6 | -42 | -46 | Brain-Stem (85.1%) |
| 119 | 4.46 | 18 | 12 | 18 | Right Caudate (9.3%); Right Putamen (50.7%) |
| 51 | 3.99 | -16 | 12 | 2 | Left Putamen (61.7%) |
| 47 | 3.99 | -8 | -36 | -4 | Brain-Stem (27.8%) |
| 33 | 4.01 | -18 | 14 | 16 | Left Lateral Ventricle (11.0%); Left Caudate (31.1%) |
| 1247† | 6.03 | 20 | -64 | -18 | Right V (5.5%); Left VI (33.8%); Right VI (33.5%); Left Crus I (6.4%); Right Crus I (7.4%) |
| 206† | 4.68 | 26 | -66 | -46 | Right VIIb (34.0%); Right VIIIa (48.2%) |
| 98† | 5.01 | -28 | -62 | -52 | Left VIIb (32.9%); Left VIIIa (51.7% |

**Supplementary Table 10** Detailed description of clusters of activation for the verbal working memory, linear encoding paradigm showing the activation cluster size, maximum intensity, coordinates of voxel with maximum intensity for clusters. Anatomical localisation based on overlap between the cluster and cortical/subcortical/cerebellar probabilistic atlases present in FSL. †Obtained using a cerebellar mask (see Methods). Results from mixed effects modelling were obtained using a cluster forming threshold of Z>3.09 and corrected P<0.05.

| **Voxels** | **Peak intensity max voxel** | **Max X** | **Max Y** | **Max Z** | **Atlas landmarks** |
| --- | --- | --- | --- | --- | --- |
| 14478 | 6.2 | -28 | -76 | 20 | Lateral Occipital Cortex, superior division (13.1%); Lateral Occipital Cortex, inferior division (7.5%); Occipital Fusiform Gyrus (6.9%) |
| 1654 | 6.06 | 50 | 6 | 40 | Superior Frontal Gyrus (5.5%); Middle Frontal Gyrus (11.9%); Inferior Frontal Gyrus, pars opercularis (5.9%); Precentral Gyrus (27.8%) |
| 1222 | 6.1 | -50 | 4 | 40 | Middle Frontal Gyrus (8.5%); Inferior Frontal Gyrus, pars opercularis (9.0%); Precentral Gyrus (36.2%) |
| 890 | 5.28 | -2 | 6 | 56 | Juxtapositional Lobule Cortex (formerly Supplementary Motor Cortex) (14.7%); Paracingulate Gyrus (34.7%); Cingulate Gyrus, anterior division (17.4%) |
| 848 | 4.64 | 22 | 14 | -4 | Left Caudate (10.2%); Left Putamen (5.0%); Right Lateral Ventricle (5.1%); Right Thalamus (5.8%); Right Caudate (17.1%); Right Putamen (18.7%) |
| 459 | 4.98 | 34 | 44 | 26 | Frontal Pole (54.1%); Middle Frontal Gyrus (13.8%) |
| 155 | 4.26 | 36 | 18 | 10 | Insular Cortex (27.6%); Frontal Operculum Cortex (31.1%) |
| 142 | 4.54 | -60 | -18 | 28 | Postcentral Gyrus (53.4%); Supramarginal Gyrus, anterior division (9.5%); Central Opercular Cortex (8.3%) |
| 123 | 4.44 | -42 | -38 | 44 | Postcentral Gyrus (15.6%); Superior Parietal Lobule (14.3%); Supramarginal Gyrus, anterior division (19.2%); Supramarginal Gyrus, posterior division (6.6%) |
| 78 | 4.11 | 2 | -36 | -4 | Brain-Stem (51.7%) |
| 61 | 4.57 | -54 | -42 | 28 | Superior Temporal Gyrus, posterior division (6.3%); Supramarginal Gyrus, posterior division (22.1%); Parietal Operculum Cortex (12.7%); Planum Temporale (16.0%) |
| 49 | 4.09 | 4 | -36 | 24 | Cingulate Gyrus, posterior division (40.7%) |
| 36 | 3.93 | -36 | 38 | 24 | Frontal Pole (58.2%); Middle Frontal Gyrus (12.8%) |
| 32 | 4.56 | -32 | 16 | 8 | Insular Cortex (41.2%); Frontal Operculum Cortex (13.9%) |
| 29 | 4.14 | -12 | -64 | 64 | Superior Parietal Lobule (18.3%); Lateral Occipital Cortex, superior division (34.2%); Precuneous Cortex (13.4%) |
| 25 | 4.02 | -18 | 18 | -10 | Left Putamen (74.1%) |
| 24 | 4.3 | 52 | 14 | -6 | Inferior Frontal Gyrus, pars opercularis (7.9%); Temporal Pole (28.8%); Frontal Operculum Cortex (6.3%) |
| 23 | 3.85 | 22 | -26 | -6 | Right Thalamus (19.0%) |
| 735 | 4.63 | 8 | -70 | -22 | Left VI (16.4%); Vermis VI (9.7%); Right VI (35.5%); Left Crus I (5.3%); Right Crus I (9.5%); Vermis Crus II (5.6%) |
| 47 | 4.34 | 26 | -64 | -48 | Right VIIb (50.3%); Right VIIIa (41.8%) |
| 39 | 4.26 | -28 | -68 | -52 | Left VIIb (62.6%); Left VIIIa (35.4%) |
| 30 | 4.17 | -24 | -38 | -42 | Left IX (5.6%); Left X (42.1%) |
| 25 | 3.97 | 0 | -52 | -36 | Vermis VIIIb (7.5%); Vermis IX (64.8%); Vermis X (23.4%) |
| 18 | 3.87 | -36 | -54 | -30 | Left VI (45.3%); Left Crus I (54.7%) |
| 14 | 3.86 | 16 | -46 | -44 | Right VIIIb (8.7%); Right IX (16.4%); Right X (27.0%) |

**Supplementary Table 11** Detailed description of clusters of activation for the showing the verbal working memory, linear maintenance paradigm showing the activation cluster size, maximum intensity, coordinates of voxel with maximum intensity for clusters. Anatomical localisation based on overlap between the cluster and cortical/subcortical/cerebellar probabilistic atlases present in FSL. †Obtained using a cerebellar mask (see Methods). Results from mixed effects modelling were obtained using a cluster forming threshold of Z>3.09 and corrected P<0.05.

| **Voxels** | **Peak intensity max voxel** | **Max X** | **Max Y** | **Max Z** | **Atlas landmarks** |
| --- | --- | --- | --- | --- | --- |
| 373 | 4.59 | 2 | 26 | 46 | Superior Frontal Gyrus (14.8%); Paracingulate Gyrus (46.7%); Cingulate Gyrus, anterior division (7.6%) |
| 257 | 4.4 | -38 | 22 | 0 | Insular Cortex (15.6%); Inferior Frontal Gyrus, pars opercularis (6.5%); Frontal Orbital Cortex (16.0%); Frontal Operculum Cortex (15.7%) |
| 225 | 4.32 | -52 | 14 | 30 | Middle Frontal Gyrus (34.3%); Inferior Frontal Gyrus, pars opercularis (15.9%) |
| 154 | 4.22 | 36 | 24 | -6 | Insular Cortex (16.3%); Frontal Orbital Cortex (28.4%); Frontal Operculum Cortex (14.6%) |
| 87 | 4.64 | -38 | 0 | 60 | Middle Frontal Gyrus (38.5%); Precentral Gyrus (15.3%) |
| 42 | 4.39 | 38 | -52 | 46 | Superior Parietal Lobule (26.7%); Angular Gyrus (27.5%) |
| 39 | 4.39 | -34 | -56 | 44 | Superior Parietal Lobule (28.2%); Supramarginal Gyrus, posterior division (9.2%); Angular Gyrus (14.7%); Lateral Occipital Cortex, superior division (14.7%) |
| 29 | 4.08 | 6 | 38 | 22 | Paracingulate Gyrus (52.9%); Cingulate Gyrus, anterior division (28.1%) |
| 29 | 4.15 | -48 | -48 | 46 | Supramarginal Gyrus, posterior division (37.7%); Angular Gyrus (14.7%) |
| 237 | 4.3 | 28 | -66 | -28 | Right VI (30.9%); Right Crus I (57.3%) |
| 95 | 4.23 | 30 | -62 | -50 | Right Crus II (12.8%); Right VIIb (62.3%); Right VIIIa (18.9%) |
| 90 | 4.18 | -4 | -82 | -26 | Lingual Gyrus (6.2%); Left VI (11.5%); Vermis VI (9.4%); Right VI (9.7%); Left Crus I (18.3%) |
| 88 | 4.13 | -36 | -62 | -28 | Left VI (5.1%); Left Crus I (90.9%) |
| 28 | 3.91 | -50 | -60 | -34 | Left Crus I (84.8%) |
| 25 | 3.97 | -38 | -60 | -40 | Left Crus I (24.9%); Left Crus II (49.2%); Left VIIb (7.5%) |

**Table 12** Summary of maximal overlap frequency and anatomical locations for frequency maps. The number in brackets under locations columns is the corresponding frequency for the reported maximal voxel. Note: the input data used when generating frequency maps for the Sternberg task was based on the parametric model during the maintenance phase.

| **Paradigm/contrast** | **Maximum frequency** | **Location of maximal voxel cerebrum** | **Location of maximal voxel cerebellum** |
| --- | --- | --- | --- |
| Motor (fingers>toes) | 20 | (20) left pre- / post-central gyrus | (17) right lobule V |
| Motor (toes>fingers) | 19 | (19) L. pre-central gyrus | (17) right lobules I-IV |
| Language (L1) | 11 | (11) right intra-calcarine cortex / occipital pole / lingual gyrus | (8) right Crus I |
| Speech motor (SM1) | 14 | 14 right pre- / post-central gyrus | (8) right lobule VI |
| Sternberg (encoding) | 18 | (18) left intra-calcarine cortex / occipital pole / lingual gyrus | (6) right lobule VI |
| Sternberg (maintenance) | 7 | (7) right middle frontal gyrus | (6) bilateral Crus I |
| Vibrotactile (finger>toe) | 8 | (8) left post-central gyrus | (2) right lobules V/VI |
| Vibrotactile (toe>finger) | 4 | (4) left post-central gyrus | (2) right lobule VI |
