# Supplementary material for: Sensorimotor, language, and working memory representation within the human cerebellum
Source: Hum Brain Mapp. 2019 Jul 30;40(16):4732–47. doi: 10.1002/hbm.24733 (PMC6865458; doi:10.1002/hbm.24733)
Supplement: Supplementary file 2 — Figure S1 Five language contrasts (versus rest). In response to the separate portions of the language task activity was seen across cortical (predominantly left sided, including Broca's and Wernicke's) and sub‐cortical regions in the cerebrum and in the cerebellum (predominantly right sided). Labelled activity is as follows: HG, Heschl's gyrus; PCG, paracingulate gyrus; CGpre, precentral gyrus; CGpo, postcentral gyrus; IFGpo, inferior frontal gyrus, pars opercularis; IFGpt, inferior frontal gyrus, pars triangularis; STGpd, superior temporal gyrus, posterior division; PT, planum temporale. Activation was determined using a cluster forming threshold of Z > 3.09 and cluster corrected p < 0.05 for the whole brain (left hand side) and for the cerebellum alone (right hand side) using a mask, see Methods. [file HBM-40-4732-s002.docx]

Supplementary Figure


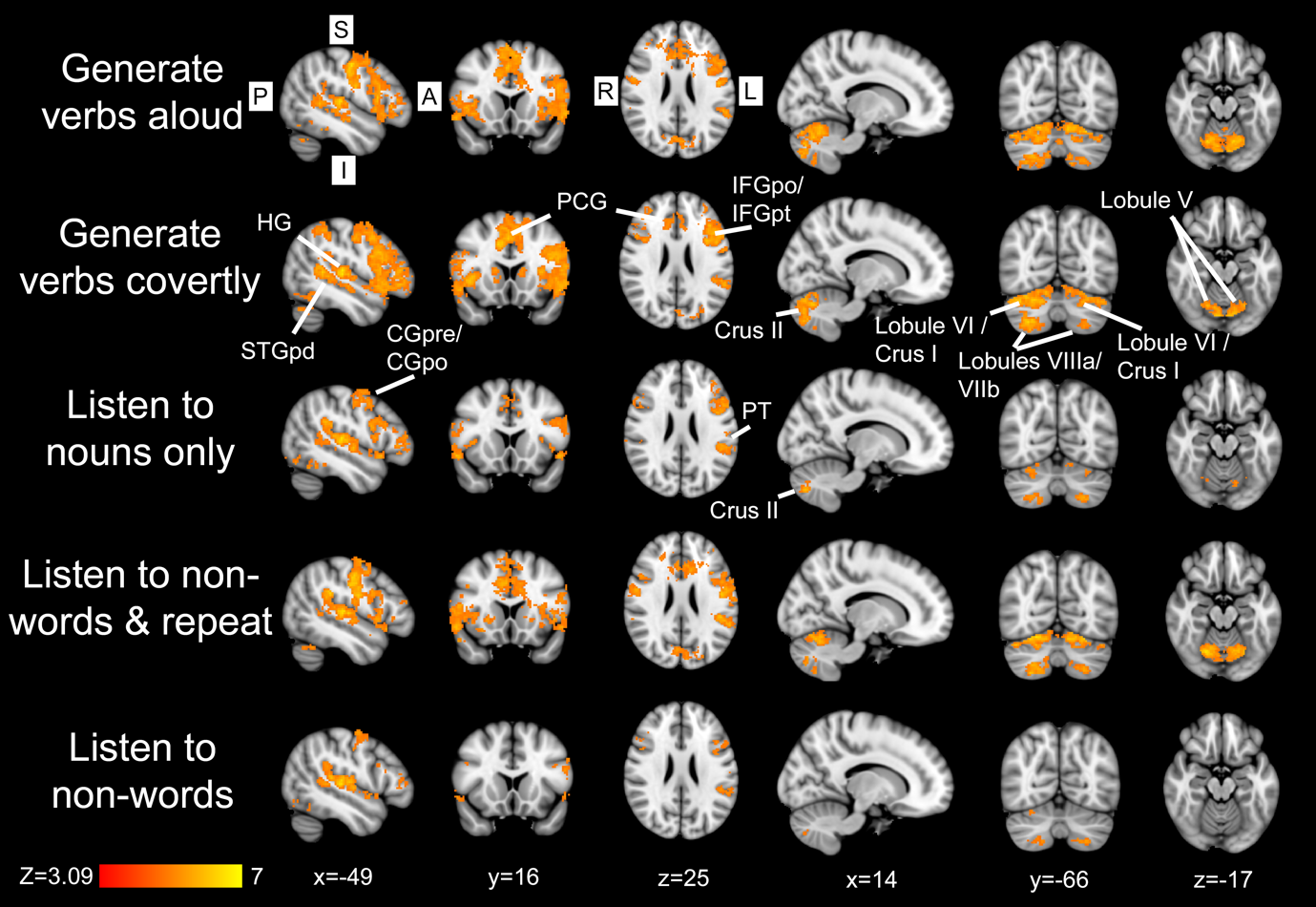


**Figure**: Five language contrasts (versus rest). In response to the separate portions of the language task activity was seen across cortical (predominantly left sided, including Broca’s and Wernicke’s) and sub-cortical regions in the cerebrum and in the cerebellum (predominantly right sided). Labelled activity is as follows: HG, Heschl’s gyrus; PCG, paracingulate gyrus; CGpre, precentral gyrus; CGpo, postcentral gyrus; IFGpo, inferior frontal gyrus, pars opercularis; IFGpt, inferior frontal gyrus, pars triangularis; STGpd, superior temporal gyrus, posterior division; PT, planum temporale. Activation was determined using a cluster forming threshold of Z>3.09 and cluster corrected P<0.05 for the whole brain (left hand side) and for the cerebellum alone (right hand side) using a mask, see Methods.
